# Consistency of resting-state correlations between fMRI networks and EEG band power

**DOI:** 10.1162/IMAG.a.37

**Published:** 2025-06-18

**Authors:** Marta Xavier, Inês Esteves, João Jorge, Rodolfo Abreu, Anne-Lise Giraud, Sepideh Sadaghiani, Jonathan Wirsich, Patrícia Figueiredo

**Affiliations:** ISR-Lisboa and Department of Bioengineering, Instituto Superior Técnico, Universidade de Lisboa, Lisbon, Portugal; CSEM—Swiss Center for Electronics and Microtechnology, Bern, Switzerland; Coimbra Institute for Biomedical Imaging and Translational Research (CIBIT), ICNAS, University of Coimbra, Coimbra, Portugal; Institut de l’Audition, Institut Pasteur, Université de Paris, INSERM, Paris, France; Department of Psychology, University of Illinois at Urbana-Champaign, Urbana, IL, United States; Beckman Institute for Advanced Science and Technology, Urbana, IL, United States; EEG and Epilepsy Unit, University Hospitals and Faculty of Medicine of Geneva, University of Geneva, Geneva, Switzerland; Swiss Center for Affective Sciences, University of Geneva, Geneva, Switzerland

**Keywords:** EEG-fMRI, resting-state networks, band power, hemodynamic response, temporal correlation, intra- and inter-dataset consistency

## Abstract

Several simultaneous electroencephalography (EEG)-functional magnetic resonance imaging (fMRI) studies have aimed to identify the relationship between EEG band power and fMRI resting-state networks (RSNs) to elucidate their neurobiological significance. Although common patterns have emerged, inconsistent results have also been reported. This study aims to explore the consistency of these correlations across subjects and to understand how factors such as the hemodynamic response delay and the use of different EEG data spaces (source/scalp) influence them. Using three distinct EEG-fMRI datasets, acquired independently on 1.5T, 3T, and 7T MRI scanners (comprising 42 subjects in total), we evaluate the generalizability of our findings across different acquisition conditions. We found consistent correlations between fMRI RSN and EEG band power time series across subjects in the three datasets studied, with systematic variations with RSN, EEG frequency band, and hemodynamic response function (HRF) delay, but not with EEG space. Several of these correlations were consistent across the three datasets, despite important differences in field strength and resting-state conditions. These included spatially widespread patterns observed across HRF delays from 2 to 10 s, such as positive delta correlations with the visual and somatomotor networks, negative delta correlations with the default mode network, positive theta correlations with the somatomotor network, negative alpha correlations with both the visual and dorsal attention networks, positive alpha correlations with the default mode network, and negative beta correlations with the somatomotor network. Our findings support consistent correlations across specific fMRI RSNs and EEG bands and highlight the importance of methodological considerations in interpreting them that may explain conflicting reports in the existing literature.

## 1 Introduction

Functional brain networks are defined as a set of distributed brain regions that are functionally interconnected, contributing to specific cognitive functions with different levels of complexity (Friston, 1994;Park & Friston, 2013;Power et al., 2011;Seeley et al., 2007;Smith et al., 2009). The study of these networks has been crucial for understanding healthy brain function, as well as identifying markers of abnormal brain activity in pathology (J. Zhang et al., 2021). Resting-state studies have been particularly useful in characterizing functional networks because they reveal intrinsic patterns of neuronal integration without the need for a specific stimulation or task paradigm. Since these resting-state patterns were first reported (Biswal et al., 1995;Raichle et al., 2001), several highly reproducible resting-state networks (RSNs) have been identified (Smith et al., 2009;Yeo et al., 2011) that are spatially consistent across different subjects as well as acquisition conditions. Over the years, blood-oxygen-level-dependent (BOLD) functional magnetic resonance imaging (fMRI) has been the preferred imaging modality for characterizing RSNs because of its high spatial resolution, whole-brain coverage, and non-invasiveness. However, the prominent non-neuronal contributions to BOLD signals, such as heart rate and respiration, may significantly confound the neuronal origin of RSNs (Keilholz, 2014;Logothetis et al., 2001). Leveraging the more direct measure of neuronal activity provided by electroencephalography (EEG), simultaneous EEG-fMRI studies have sought to identify electrophysiological correlates of fMRI RSNs (as highlighted in multiple reviews, e.g.:Abreu et al., 2018;Murta et al., 2015;Chang & Chen, 2021;Ciccarelli et al., 2023;Fleury et al., 2023;Jorge et al., 2014;Wirsich et al., 2023).

Early EEG-fMRI studies typically used multiple regression frameworks to combine average EEG activity from a set of mostly occipital electrodes, filtered in one or a few specific frequency bands, with voxel-wise whole-brain BOLD activity (Goldman et al., 2002;Gonçalves et al., 2006;Laufs et al., 2003a,2003b,^2006^;Moosmann et al., 2003). Most of these focused on the alpha frequency band (8–12 Hz) and reported solely negative alpha–BOLD correlations. Later research began to identify RSNs explicitly, often through independent component analysis (ICA) or seed-based linear correlation and sought to correlate these networks’ BOLD activity with EEG frequency band power. A key study byMantini et al. (2007)demonstrated that canonical RSNs (obtained with ICA) could each be characterized by specific combinations of EEG frequency bands. Notably, they found significant positive correlations between alpha power (8–13 Hz) and the default mode network (DMN), contrasting with the literature that had reported mostly negative alpha–BOLD correlations.

Subsequent attempts to replicate these findings found large variations between subjects leading to non-significant group results (Meyer et al., 2013), raising questions about individual differences. Other studies have suggested that rest conditions (e.g., eyes-open vs. eyes-closed) (Mo et al., 2013), spatial inhomogeneities within EEG frequency band power (Jann et al., 2010;Scheeringa et al., 2008), and fMRI RSNs (Bowman et al., 2017;Marawar et al., 2017), as well as time-varying changes (Mayhew & Bagshaw, 2017), contribute to the complex relationship between EEG band power and RSNs. Specifically,Mo et al. (2013)observed positive correlations between alpha power and the DMN during eyes-open rest, but not eyes-closed rest.Jann et al. (2010)demonstrated that band-specific EEG power co-varied with fMRI RSNs in distinct clusters of channels and frequency bands.Scheeringa et al. (2008)found negative correlations between a frontal midline theta component (obtained with spatial ICA) and regions within the DMN.Bowman et al. (2017)further highlighted spatial inhomogeneities within the DMN itself, reporting both positive and negative correlations between alpha power and different DMN sub-networks (obtained with spatial ICA). Similarly,Marawar et al. (2017)found both positive and negative EEG–BOLD correlations for the delta and theta bands across different DMN regions. Additionally,Mayhew and Bagshaw (2017)demonstrated that alpha–BOLD correlations are dynamically modulated over time, suggesting that static analyses may fail to capture the full complexity of these interactions.

A variety of other approaches have also been employed over the years to investigate the relationship between EEG-derived signals and fMRI RSNs. For instance, some have investigated EEG microstates (Britz et al., 2010;Musso et al., 2010;Rajkumar et al., 2021;Schwab et al., 2015), EEG ICs (Bridwell et al., 2013;Hiltunen et al., 2014;Labounek et al., 2019;Prestel et al., 2018;Yuan et al., 2012), source-localized EEG spectral features (Neuner et al., 2014), spatial-temporal-spectral EEG patterns (Lamoš et al., 2018), and nonlinear EEG measures (Portnova et al., 2018), while others explored dynamic functional connectivity in fMRI (Allen et al., 2018;Chang et al., 2013;Phadikar et al., 2025;Tagliazucchi et al., 2012) or applied machine learning-based predictions (Abreu et al., 2021;Meir-Hasson et al., 2014;Simões et al., 2020).

The previous paragraphs highlight the wide range of methodological heterogeneities that characterizes studies investigating EEG-fMRI RSN relationships. These can be in terms of EEG features considered (e.g., different frequency bands, electrode subsets, or more complex measures such as microstates and non-linear metrics), fMRI network identification (e.g., varying approaches such as ICA, seed-based correlation, spatial templates), integration strategies (e.g., multiple regression frameworks, temporal correlations, machine-learning-based predictors). Beyond these analytical choices, methodological heterogeneities also arise from variations in study design (e.g., number of participants, eyes-open vs. eyes-closed conditions, field strength, fMRI acquisition sequence parameters, namely in terms of spatial and temporal resolution, EEG system used, namely in terms of number of channels) as well as differences in data preprocessing. This great heterogeneity found in the literature is clearly illustrated by the details of the studies presented inTable 1andSupplementary Table S1.

**Table 1.** Studies that investigate the temporal correlation between fMRI-derived resting-state networks (RSNs) and EEG spectral measures.

						RESULTS	
Study	Study design	EEG measures	EEG freq. bands (Hz)	fMRI measures	Integration	Positive relationship	Negative relationship
Mantini et al., 2007	15 subjects Rest, EC1.5T MR scanner 32 EEG channels	Band-specific power TS; HRF conv. (6-s peak); Avg. across all channels	δ (1-4) θ (4-8) α (8-13) β (13-30) γ (30-50)	sICA – IC TS (DMN)	Univariate Pearson’s correlation	DMN: δ, θ, α, β, γ	VN: δ, θ, α, β, γ SMN: δ, θ, α, β, γ DAN: δ, θ, α, β, γ
Jann et al., 2010	14 subjects Rest, EC 3T MR scanner 92 EEG channels	Band-specific power TS; 4-6 s delay; All channels	δ (1.0-3.5) θ-1 (3.5-6.25) θ-2 (6.25-8.2) α-1 (8.2-10.5) α-2 (10.5-14.0) β-1 (14.0-18.75) β-2 (18.75-21.88) β-3 (21.88-30)	sICA – IC TS (VN, SMN, FPN, DMN)	Univariate covariance	VN: δ, θ (occ.); SMN: δ (cent.) FPN: α-1, α-2, β-1, β-2 (occ.) DMN: α-1 (central); α-2 (par-occ.); β-1 (par.)	VN: α-1, α-2, β-1, β-2 (occ., par.) SMN: α-1, α-2 (cent-par.), β-1, β-2 (cent-par.) FPN: α-2 (front.), θ (cent-occ.)DMN: δ, θ-1, θ-2 (fronto-cent., par-occ.)
Sadaghiani et al., 2010	26 subjects Rest, EC3T MR scanner 62 EEG channels	Band-specific power TS; HRF conv. (6-s peak, SPM); All channels	low α (7-10)upper α (9-12) low β (15-18)broad β (17-24)	Seed-based regression RSN TS (TAN, DAN)	Multivariate regression	TAN: upper α, broad β	DAN: low α, low β
Meyer et al., 2013	12 subjects Rest, EO 3T MR scanner30 EEG channels	Band-specific power TS; HRF conv. (6-s peak, SPM5); Avg. across all channels	δ (2-4) θ (4-7) α (8-12) β (12-30)	sICA – IC TS (VN, SMN, FPN, DMN)	Univariate Pearson’s correlation	VN: δ, θ, β SMN: δ, θ FPN: δ DMN: δ, θ	VN: α SMN: α, β FPN: α, β, θ DMN: α, β
Bowman et al., 2017	20 subjects Rest, EC 3T MR scanner 62 EEG channels	Band-specific power TS; HRF conv. (6-s peak, SPM); Avg. across P3-O1, P4-O2, P7-O1, P8-O2	α (8-13)	sICA – IC TS (subnetworks of DMN)	Univariate Pearson’s correlation	DMN: α (PCC + mPFC + precuneus)	DMN: α (PCC + parietal; sup. front. + ACC; medial frontal + bilateral temporal)

These methodological heterogeneities, often present in more than one aspect, have potentially contributed to the inconsistencies in the findings reported in the literature and the difficulty of interpreting them. In fact, EEG data recorded in the scanner are inherently noisy and any EEG features extracted are strongly dependent on the artifact removal procedure. Critically, some frequencies tend to be more corrupted than others and, therefore, the choice of frequencies analyzed may also impact the results. For instance,Fellner et al. (2016)showed that EEG signals below 20 Hz are strongly correlated with in-scanner motion, which can cause spurious correlations and contribute to variability between subjects and sessions. Moreover, since the EEG spectral power is correlated across different frequency bands, results may differ depending on whether univariate or multivariate regression frameworks are employed (de Munck et al., 2009). Most strikingly, even studies that employed similar data integration strategies have occasionally reported contradictory results—as exemplified inTable 1. This raises the question of whether inconsistencies are due mainly to methodological differences in data analysis or also to differences in data quality and acquisition setup and even between subjects.

A further methodological consideration is the choice of hemodynamic response function (HRF). Although most previous studies rely on a canonical HRF, it is known to vary across regions, individuals, and conditions (Logothetis & Wandell, 2004). For instance,de Munck et al. (2007)showed that alpha band-related BOLD fluctuations can exhibit regional variability in the HRF, influencing EEG–fMRI correlation results. Likewise, the use of scalp-referenced EEG data, which is prone to volume conduction effects and has limited spatial specificity, may obscure the direct correspondence between electrophysiological signals and localized BOLD activity.

To address these complexities, our study applies a unified data analysis pipeline to multiple independently acquired resting-state EEG-fMRI datasets, each with different acquisition setups. By focusing on a comparable EEG–fMRI integration method—namely, correlating data-driven fMRI RSNs with EEG spectral power—we aim to systematically evaluate the consistency of these relationships within and across datasets.Table 1summarizes the main findings from studies that adopted a similar integration method, whileSupplementary Table S1offers a more extensive overview of all reported EEG power correlates of BOLD RSN activity (or BOLD activity within an RSN) during rest. AlthoughTable 1shows some recurring observations—for instance, negative correlations between alpha/beta power and the sensorimotor network, negative correlations between alpha power and the VN—there remain notable inconsistencies, such as both positive and negative delta/theta correlations with the SMN (Jann et al., 2010;Mantini et al., 2007;Meyer et al., 2013), as well as both positive and negative, or even variable, alpha/beta correlations with the FPN (Jann et al., 2010;Meyer et al., 2013). Variable correlations are reported across frequency bands and the DMN (Bowman et al., 2017;Jann et al., 2010;Mantini et al., 2007;Meyer et al., 2013), while other canonical RSNs, such as the dorsal and ventral attention networks (DAN, VAN), are inconsistently present in the studies.

Accordingly, our study expands on previous approaches in several important ways. First, we systematically vary the HRF to assess whether, and to what extent, HRF variability influences EEG–fMRI correlations. Second, we incorporate source-space EEG data to explore whether this additional spatial specificity alters or clarifies observed relationships with BOLD RSNs.

## 2 Materials and Methods

### 2.1 Datasets

Three previously acquired and reported simultaneous EEG-fMRI datasets were used in this study. The respective subject groups and acquisition setups are described below.

#### 2.1.1 Dataset 1 (1.5T)

Simultaneous EEG-fMRI data were acquired from 16 healthy volunteers, during one session of 10 min 48 s eyes-open resting state. Of the 16 subjects, only 10 were selected for inclusion in this study based on the consistency of their EEG electrode placements, ensuring spatial uniformity within the dataset. Ethical approval was given by the local research ethics committee (UCL Research ethics committee; committee, project ID: 4290/001) and informed consent was obtained from all subjects (Deligianni et al., 2014,^2016^). This dataset is publicly available from the Open Science Framework (OSF;https://osf.io/94c5t/). Subjects were asked to avoid movement, remain awake, and fixate on a white cross presented on a black background. MRI data were acquired using a 1.5T Siemens Avanto scanner equipped with a self-shielded gradient set (maximum gradient amplitude: 40 mT/m) and a standard 12-channel head receiver coil. fMRI was acquired with a T2∗-weighted gradient-echo echo-planar imaging (GRE-EPI) sequence with 300 volumes and the following parameters: TR/TE = 2160/30 ms, flip angle 75°, 30 slices (slice thickness 3.0 mm and 1 mm gap), field of view (FOV) = 210 × 210 × 120 mm^3^, and voxel size = 3.3 × 3.3 × 3.0 mm^3^. A T1-weighted structural image was also acquired (176 sagittal slices, voxel size = 1.0 mm isotropic). Scalp EEG was recorded with two 32-channel MR-compatible amplifiers (BrainAmp MR, sampling rate 1 kHz) and 63 electrodes (BrainCap MR). The electrodes were arranged according to the modified combinatorial nomenclature, referenced to FCz, and 1 ECG electrode was used. The EEG amplifiers were time locked with the scanner clock.

#### 2.1.2 Dataset 2 (3T)

Simultaneous EEG-fMRI was acquired from 26 healthy volunteers, during 3 consecutive runs of 10 min eyes-closed resting state. Out of the 26 subjects, only 23 were selected for inclusion in this study, specifically those for whom complete data from all three runs were available for both EEG and fMRI. Ethical approval was given by the local research ethics committee (CPP Ile-de France III) and informed consent was obtained from all subjects (Morillon et al., 2010;Sadaghiani et al., 2010). Subjects were asked to avoid movement and remain awake. MRI data were acquired using a 3T Siemens Tim-Trio scanner with a standard 12-channel head coil. fMRI was acquired with a GRE-EPI sequence with 300 volumes (900 total for all runs) and the following parameters: TR/TE = 2000/50 ms, flip angle 78º, 40 slices, FOV = 192 × 192 × 120 mm^3^, and voxel size = 3.0 × 3.0 × 3.0 mm^3^. A T1-weighted structural image was also acquired (176 sagittal slices, FOV = 256 × 256 mm^2^, voxel size = 1.0 mm isotropic). Scalp EEG was recorded with two 32-channel MR-compatible amplifiers (BrainAmp MR, sampling rate 5 kHz) and 62 electrodes (Easycap electrode cap). The electrodes were referenced to FCz, and two additional electrodes, one ECG and one EOG, were used. The EEG amplifiers were time locked with the scanner clock.

#### 2.1.3 Dataset 3 (7T)

Simultaneous EEG-fMRI was acquired from nine healthy volunteers, during one session of 8 min eyes-open resting state. Ethical approval was given by the local research ethics committee (CER-VD) and informed consent was obtained from all subjects (Jorge et al., 2019). Subjects were asked to avoid movement, remain awake, and fixate on a red cross presented on a gray background. MRI data were acquired using a 7T/68 cm actively shielded Siemens Magnetom scanner with an 8-channel head RF array (Rapid Biomedical). fMRI was acquired with a 2D simultaneous multi-slice (SMS) gradient-echo EPI sequence (3 × SMS acceleration with shift factor = 3, and 2 × in-plane GRAPPA acceleration) with 480 volumes and the following parameters: TR/TE = 1000/25 ms, flip angle 54º, 69 slices, voxel size = 2.2 × 2.2 × 2.2 m^3^. An EPI image with reversed phase encoding direction was also acquired (five volumes) in order to perform image distortion correction. A T1-weighted structural image was acquired with a 3D gradient-echo MP2RAGE sequence (160 sagittal slices, voxel size = 1.0 mm isotropic). Scalp EEG was recorded with two 32-channel MR-compatible amplifiers (BrainAmp MR, sampling rate 5 kHz) and 63 electrodes (Easycap electrode cap). The electrodes were referenced to FCz, and one additional ECG electrode was also used. Four of the 64 electrodes (T7, T8, F5, and F6) were modified to serve as motion artifact sensors (Jorge et al., 2015), leaving 59 electrodes for EEG recording. The EEG amplifiers were time locked with the scanner clock. Respiratory traces were also recorded (sampling rate 50 Hz) with a respiratory belt from the physiological monitoring unit of the MRI system.

### 2.2 MRI data analysis

MRI data analysis was performed using tools from the FMRIB’s Software Library (FSL 6.0.2) (Smith et al., 2004).

#### 2.2.1 MRI preprocessing

The T1-weighted structural image was first reoriented to the standard orientation and cropped to remove head and lower neck (using FSL’s tools*fslreorient2std*and*robustfov*), corrected for bias field inhomogeneities using FSL-FAST (Y. Zhang et al., 2001) and then brain extracted using FSL-BET (Smith, 2002). The structural data were then coregistered to the standard template—Montreal Neurological Institute (MNI) (Collins et al., 1994). For this, a 12 degrees of freedom (DOF) linear transformation—estimated with FSL-FLIRT (Jenkinson & Smith, 2001;Jenkinson et al., 2002)—was used to initialize a non-linear transformation—estimated with FSL-FNIRT (Andersson et al., 2007). FSL-FAST was used to segment the structural data into white matter (WM), cerebrospinal fluid (CSF), and gray matter (GM) tissues.

#### 2.2.2 fMRI preprocessing

Non-brain tissue was removed using FSL-BET, motion correction was performed with FSL-MCFLIRT (Jenkinson et al., 2002), and data were high-pass filtered using a nonlinear filter with a cutoff period of 100 s. Spatial smoothing using a Gaussian kernel with full-width at half-maximum (FWHM) of about 1.5 times the voxel size was then performed (5 mm for 1.5T, 4 mm for 3T, 3 mm for 7T) using FSL-SUSAN (Smith & Brady, 1997).

Functional data were co-registered to the structural image (using FSL-FLIRT with 12 DOF) and co-registered to the standard MNI template by applying the estimated linear transformation, followed by the non-linear transformation described above to co-register the structural image to the standard template. The following nuisance regressors were linearly regressed out of the data as follows:

(1) 24 motion realignment parameters: the 6 motion realignment parameters (RP) estimated with FSL-MCFLIRT were first temporarily high-pass filtered with the same filter used for the functional data, to avoid the reintroduction of artifactual variance in filtered frequencies. Then the following time-series expansions were computed: their temporal derivatives (estimated as the difference between the original time series and the backward-shifted time series), the quadratic term of these derivatives, and the temporal derivative of the quadratic term.

(2) Motion outliers: motion outliers were estimated with FSL’s*fsl_motion_outliers*, using the metric DVARS (Power et al., 2012), which is computed as the root mean square intensity difference of volume N to volume N + 1, normalized by the median brain intensity and multiplied by 1000. To identify outliers, the DVARS was thresholded at the 75th percentile + 1.5 times the interquartile range.

(3) WM and CSF time series: WM and CSF masks were obtained by segmentation of the T1-weighted structural image using FSL-FAST and then transformed to functional space using the transformation matrices described above and eroded with a 2.2 mm (WM mask) / 1.8 mm (CSF mask) Gaussian kernel to minimize partial volume effects. The CSF mask was additionally intersected with the mask of the large ventricles in the MNI atlas, also transformed into the functional space. The average BOLD signal time series in each of these masks was then computed.

##### 2.2.2.1 Specific strategy for dataset 7T

7T data were preprocessed according toAbreu et al. (2021). Slice timing correction and motion correction were performed with FSL’s MCFLIRT, followed by B0 distortion correction with FSL-TOPUP (Andersson et al., 2003). Nuisance regression was performed with the following additional regressors: BOLD fluctuations related to cardiac and respiratory cycles (RETROICOR,Glover et al., 2000) and with changes in heart rate, and with depth and rate of respiration (Chang et al., 2009).

#### 2.2.3 Identification of fMRI RSNs

The following procedure was applied independently to each dataset, as illustrated inFigure 1Bottom. Seven canonical RSNs were identified by group-level probabilistic spatial ICA (sICA) of the fMRI data, followed by template matching inYeo et al. (2011): visual (VN), somatomotor (SMN), dorsal attention (DAN), ventral attention (VAN)—anatomically similar to the Salience (Seeley et al., 2007) and Cingulo-Opercular networks (Dosenbach et al., 2007)—limbic (LN), frontoparietal (FPN), and default mode (DMN).

**Fig. 1.**
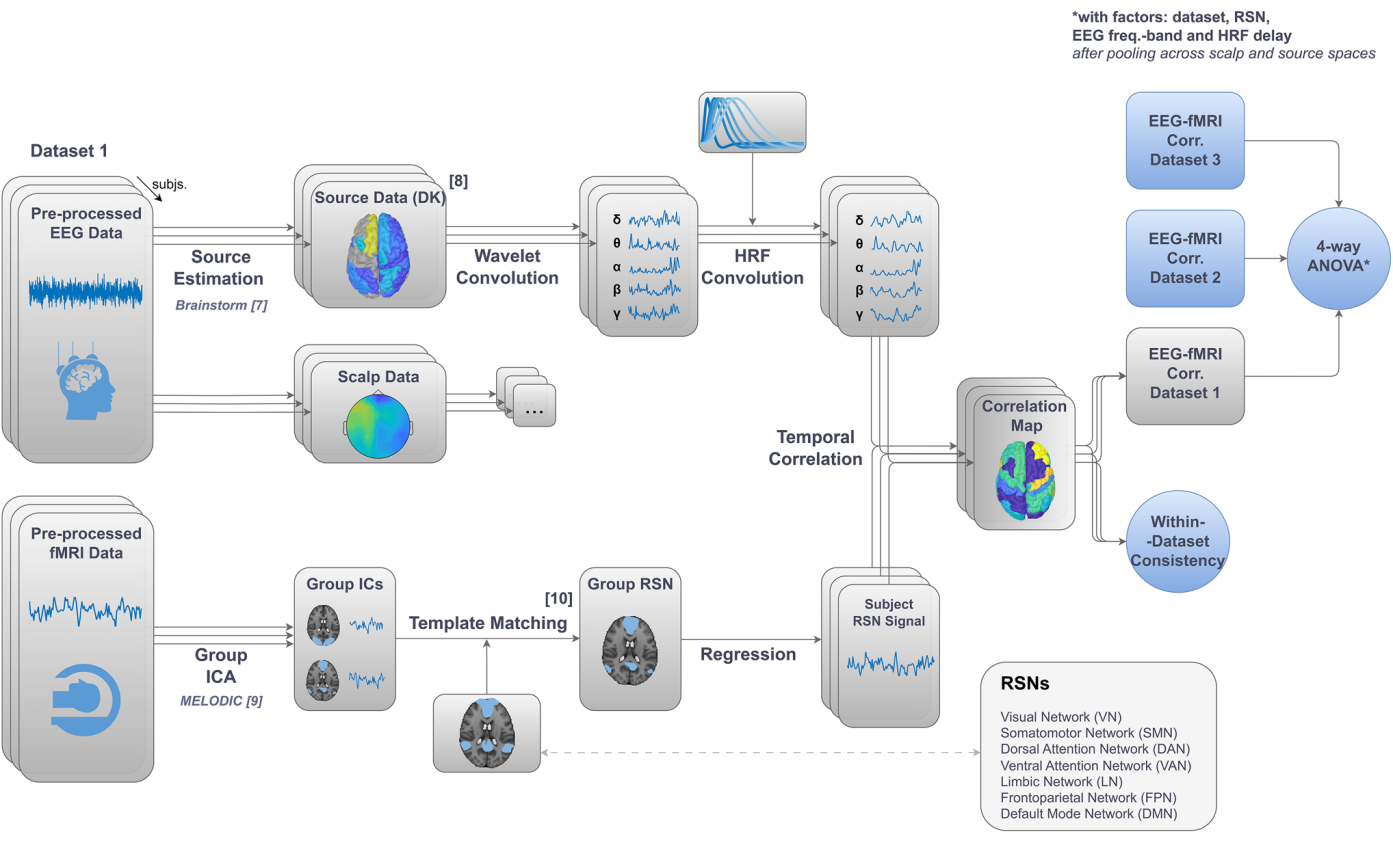
Overview of the EEG-fMRI analysis pipeline. (Bottom) For each dataset, fMRI pre-processed data underwent group-level ICA, followed by template matching (Yeo et al., 2011), in order to identify seven canonical RSNs. Subsequently, the resulting maps were regressed into each subject’s fMRI data to derive individual RSN time series. (Top) In parallel, EEG pre-processed data were subjected to source estimation to derive source activity data, which were spatially downsampled to align with the Desikan–Killiany (DK) atlas. Both scalp- and source-space EEG data underwent the following analysis: estimation of the power at each frequency band at each source node through Morlet-wavelet temporal convolution, and convolution with a family of hemodynamic response functions (HRFs) with a range of overshoot delays (2 to 10 s). (Right) The resulting EEG features of each subject were temporally correlated with the simultaneously acquired fMRI RSN time series using Pearson’s correlation. To assess consistency across subjects, t-tests against zero were conducted on spatially averaged correlation maps. Finally, a four-way repeated measures ANOVA was performed on spatially averaged correlation values (further averaged across EEG spaces via data pooling), to evaluate the impact of dataset, RSN, EEG frequency band, and HRF delay.

Group-level sICA was performed individually for each dataset, using FSL-MELODIC (Beckmann & Smith, 2004), whereby data from all subjects were temporally concatenated prior to estimating the ICs. The number of ICs was set in all datasets to be 30. The resulting ICs were associated with probabilistic spatial maps, consisting of independent spatial patterns common to all subjects in the dataset.

To link each of the canonical RSNs to a group independent component, IC statistical maps were thresholded (Z = 3) and binarized. Each canonical RSN was then automatically associated with the IC yielding the highest Dice coefficient (Dice, 1945) with the respective template. The spatial maps of the selected group ICs are displayed inSupplementary Figure S1for each dataset.

To estimate subject-level time courses for each RSN, dual regression was performed to the group-level ICs using FSL—*DualRegression*(Nickerson et al., 2017), and the time courses of the respective ICs were retrieved.

### 2.3 EEG data analysis

#### 2.3.1 Preprocessing

Different strategies were employed for each of the datasets, as described in the previous publications indicated. These are briefly described below.

**1.5T and 3T.**Data were preprocessed as described inWirsich et al. (2021). EEG was corrected for the gradient artifact using template subtraction and adaptive noise cancellation. 3T data were low-pass filtered at 75 Hz. Data were then downsampled at 250 Hz followed by pulse artifact correction with template subtraction, using the EEGLAB FMRIB plug-in (https://fsl.fmrib.ox.ac.uk/eeglab/fmribplugin/). For the 1.5T dataset, ICA-based denoising was performed in order to remove gradient and pulse artifact residuals as well as eye blinks and muscle artifacts.

**7T.**Data were preprocessed as described inJorge et al. (2015,^2019^). EEG was corrected for the gradient artifact using template subtraction. Bad-channel interpolation was performed (0–4 channels per subject, interpolated using 3–6 neighbors), followed by temporal bandpass filtering (1–70 Hz) and pulse artifact correction (using a k-means clustering approach) and downsampling at 500 Hz. Data were then corrected for motion artifacts (offline multi-channel recursive least-squares regression with the motion sensor signals), and ICA-based denoising was performed for removal of gradient and pulse artifact residuals as well as eye blinks and muscle artifacts.

**All datasets.**Time segments contaminated with motion were identified through a semi-automatic procedure, in which time points where the signal exceeded the mean channel time course by 4 std were automatically pre-selected and then visually inspected (Wirsich et al., 2020). Data were bandpass filtered at 0.5–70 Hz (1.5T), 0.3–70 Hz (3T), and 1–70 Hz (7T).

Because each dataset was acquired using different MR hardware and parameter settings (e.g., field strength) and had different EEG setups (e.g., cap geometry), a fully uniform pipeline across all datasets was neither straightforward nor necessarily optimal. Instead, we chose to preserve each dataset’s established preprocessing approach (according toJorge et al., 2015;Wirsich et al., 2021) in order to maximize data quality under its specific conditions.

#### 2.3.2 EEG source imaging

EEG data were submitted to a source imaging procedure, in order to estimate the activity at the neural sources responsible for generating the recorded electrical potential distributions on the scalp. Cortical 3-dimensional surfaces (scalp/skull, skull/brain, and CSF interfaces) were obtained with Freesurfer (Fischl, 2012,http://surfer.nmr.mgh.harvard.edu/; v7.1.0), using individual T1-weighted structural images. The remaining steps of the source imaging procedure were performed with the Brainstorm software (Tadel, 2011,http://neuroimage.usc.edu/brainstorm; version June 2022), using the recommended default options for parameters and settings.

Individual scalp surfaces representing the head–air interface were generated based on MRI structural images. The electrode positions were co-registered into the MRI structural images by a three-step procedure: initial manual adjustment of standard fiducial points, refinement by an automated algorithm, and final manual corrections to accommodate individual structural variations. Head models were estimated using individual cortical and scalp surfaces, using the OpenMEEG BEM method (Symmetric Boundary Element Method from the open source software OpenMEEG;Gramfort et al., 2010;Kybic et al., 2005). Conductivity values were assigned to the BEM compartments with defaults of 0.3 S/m for the scalp and brain compartments and 0.006 S/m for the skull compartment. In total, 15000 constrained source dipoles were placed perpendicularly to the 3D cortical surface and a lead field matrix was estimated to map all possible dipole configurations onto scalp potential distributions (forward problem). The minimum-norm (MN) method was applied to project scalp data onto the cortex (inverse problem), optimizing the fit using a regularizer to favor solutions with minimal brain activity amplitude.

Depth weighting was applied to modify the source covariance model, reducing dominance of shallower sources in MN current density maps. The signal covariance matrix was derived from the source model, incorporating orientation and depth weighting. Due to the absence of noise recordings, the noise covariance matrix was assumed as an identity matrix, implying uniform noise variance across sensors. These matrices were combined to form the data covariance matrix. The signal-to-noise ratio was set to 3, balancing the weight the signal model should be given relative to the noise model.

Source reconstructed data (15000 dipoles) were spatially averaged into the 68 regions of the Desikan–Killiany cortical atlas (Desikan et al., 2006).

#### 2.3.3 EEG feature extraction

For each subject, the time courses of the following EEG features were derived: band power (BP) in five canonical frequency bands, using Brainstorm, as illustrated inFigure 1Top. Data were segmented in epochs with the duration of one fMRI TR. All features were estimated both in the scalp and source EEG spaces, for each channel (scalp) or atlas region (source) and for each epoch.

##### 2.3.3.1 EEG band power

Time–frequency (TF) decomposition was performed by temporal convolution with complex Morlet wavelets (time resolution of 3 s at central frequency 1 Hz). The relative power of the signal at each frequency and time point was calculated as the square amplitude of the complex wavelet coefficients, averaged across the canonical EEG frequency bands (delta (2–4 Hz), theta (5–7 Hz), alpha (8–12 Hz), beta (15–29 Hz), gamma (30–60 Hz)) and normalized by the total power (1–60 Hz). The resulting EEG time series were downsampled to the fMRI TR frequency, using a finite impulse response (FIR) anti-aliasing low-pass filter. The spatial maps of the average relative power at each frequency band are displayed inSupplementary Figure S2for each dataset. Overall, these maps indicate that the spatial distributions of power are consistent across datasets, despite differences in acquisition setups and some preprocessing steps. As expected, alpha power consistently peaks in posterior regions, whereas theta power is more pronounced in frontal regions. Beta and gamma power exhibit relatively broader distributions. Notably, in the 7T dataset, we observe higher relative gamma power than the 1.5T and 3T datasets, which may reflect the increased sensitivity of higher field strengths to high-frequency signals or, alternatively, more pronounced artifacts in the gamma range.

##### 2.3.3.2 Hemodynamic response function

To account for the delay of the BOLD signal relative to the EEG, EEG features were nonlinearly transformed through convolution with a 32-s canonical HRF (defined as the combination of two gamma functions, one modeling the response peak and the other the post-stimulus undershoot), using the MATLAB toolbox SPM12 (Penny et al., 2007). Due to the known variability of the hemodynamic response across subjects and brain regions, each EEG feature was convolved with a family of HRFs, with varying shapes characterized by different overshoot delays (relative to onset): 2, 4, 5, 6, 8, and 10 s. To ensure a coherent and physiologically plausible HRF shape across different overshoot delays, the corresponding shape parameters—undershoot delay and dispersion of both overshoot and undershoot—were linearly scaled in relation to the overshoot delay, preserving the dynamics of the hemodynamic response. The time series of the resulting HRFs are displayed inSupplementary Figure S3. It is important to note, however, that this linear scaling approach simplifies the true variability of HRFs, which can differ considerably from the canonical shape (de Munck et al., 2007).

### 2.4 EEG-fMRI analysis

#### 2.4.1 Joint motion scrubbing

Both EEG epochs and fMRI volumes that were too contaminated with motion were excluded from the analysis. The fMRI volumes discarded corresponded to the motion outliers identified with FSL’s*fsl_motion_outliers*, using the criteria described above. For the EEG, epochs were discarded according to the procedure described inWirsich et al. (2020), whereby epochs containing motion artifacts were visually identified, after pre-selecting epochs where the signal in any channel exceeded its mean by at least 4 standard deviations.

Although both EEG and fMRI motion outliers were identified during preprocessing, the removal of motion-contaminated segments was one of the last steps to be carried out. Specifically, the time–frequency decomposition and subsequent HRF convolution of the derived band power signals (see Section 2.3.3.) were performed before discarding segments flagged as motion contaminated. These segments were then excluded just prior to integrating the EEG with fMRI data. While this approach may allow for minor contamination at neighboring time points, it was deemed preferable to any method that would disrupt the temporal continuity required for time–frequency analysis and HRF convolution.

The joint motion scrubbing procedure resulted in the following mean number of epochs discarded per subject: 20.7 ± 18.8 out of 295 (EEG = 18.5 ± 16.1; fMRI = 2.7 ± 4.1) for the 1.5T dataset, 49.6 ± 30.2 out of 870 (EEG = 40.6 ± 30.1; fMRI = 18.3 ± 14.1) for the 3T dataset, and 104.7 ± 40.4 out of 470 (EEG = 88.6 ± 51.1; fMRI = 27.1 ± 8.6) for the 7T dataset.

#### 2.4.2 EEG–fMRI temporal correlations

To estimate the degree of co-fluctuation of each fMRI RSN time series with each EEG feature time series, the Pearson’s correlation coefficient was computed between these signals, as illustrated inFigure 1. Right. For each subject, fMRI RSN, EEG space, EEG frequency band, and HRF overshoot delay, a correlation map was obtained, in which EEG–fMRI correlations are displayed at each channel (scalp space) or Desikan node (source space). Average dataset correlation maps were obtained by averaging the subject-specific correlation maps across all the subjects of each dataset. Finally, grand-average maps across datasets were obtained by averaging the subject-specific correlation maps across the subjects of all datasets. Since datasets used different channel configurations, the subject-averaged scalp maps were obtained for only the 48 channels that were common to all datasets.

#### 2.4.3 Statistical analyses

To assess the consistency of the correlations obtained across subjects in each dataset, two-sided t-tests were conducted against a null hypothesis of zero on the spatially averaged correlation maps, for each RSN, frequency band, and delay. Spatial averaging of correlation values, either across all channels in scalp space or across all Desikan atlas regions in source space, was employed as a strategy for dimensionality reduction. This decision was supported by the observation that correlations did not in general display polarized patterns (i.e., exhibiting both positive and negative values across different scalp or source areas). This approach allows us to preserve the spatial resolution of the EEG data while simplifying statistical testing of the effects of different factors (frequency band, RSN, delay) on the (spatially averaged) correlation values. Prior to conducting t-tests, the normality of the data was verified using the Shapiro–Wilk test. Additionally, two-sided t-tests were employed on the spatially averaged correlation maps across the subjects of all datasets. To avoid false positives, the significance p-value threshold was adjusted for multiple comparisons (across RSNs, frequency bands, and HRF delays) by employing the False Discovery Rate (FDR) correction. Both corrected and uncorrected effects are reported.

To evaluate the impact of various factors on the spatially averaged EEG–fMRI correlation values, a five-way repeated measures ANOVA was conducted using JASP (available athttps://jasp-stats.org/). The factors included in the analysis were dataset, RSN, EEG space (source/scalp), EEG frequency band, and HRF delay, with the spatially averaged EEG–fMRI correlation values used as the dependent variable and subjects being treated as a random factor. Significant effects identified in the ANOVA were further explored using post hoc tests, with Bonferroni correction to adjust for multiple comparisons, in order to identify specific differences among the levels of significant factors or interactions. Since no significant main effect or interactions (p > 0.05) were found for the EEG space, a data pooling strategy was implemented by averaging scalp- and source-space data. This was performed in order to preserve data from both types of EEG spaces, enhancing the generalizability of the findings. Hence, a four-way repeated measures ANOVA was then performed with dataset, RSN, EEG frequency band, and HRF delay as independent variables.

#### 2.4.4 Complementary analyses

Given the inherent increase in the statistical power of t-tests with the number of observations (here, the number of subjects), it is crucial to examine its effects on the presented results. Indeed, the investigated datasets exhibit significant differences in the number of subjects, with 10 subjects in the 1.5T dataset, 23 in the 3T dataset, and 9 in the 7T dataset, which could potentially impact the estimated consistency of EEG–fMRI correlations for each dataset. Moreover, the datasets also present substantial variations in scan duration (10 min for the 1.5T dataset, 30 min for the 3T dataset, and 8 min for the 7T dataset). While this does not directly impact the t-test statistical power, it may nevertheless influence the robustness of the temporal correlation estimation given its dependence on the number of time points. Hence, complementary analyses were performed in order to test the effect of varying the sample size and scan duration on the consistency of EEG–fMRI correlations. To do so, for each dataset, segments of data were randomly selected (ranging from 1 to n consecutive minutes, over 5000 iterations) prior to computing the temporal correlations and t-stat values.

The analysis of EEG–fMRI correlations detailed in the sections above focused on a set of HRF delays from 2 to 10 s, equating to a -4 to +4 s variation around the canonical 6-s delay, typically acknowledged as physiologically relevant (Logothetis & Wandell, 2004). However, exploring a broader spectrum of HRF delays could uncover additional dynamics and lags in EEG-fMRI interactions, in particular regarding the variability of hemodynamic response across different networks and frequency bands. As such, additional EEG–fMRI correlation analyses were conducted, extending the range of HRF delays considered from 0 to 20 s.

## 3 Results

### 3.1 EEG–fMRI correlations

**Fig. 2.**
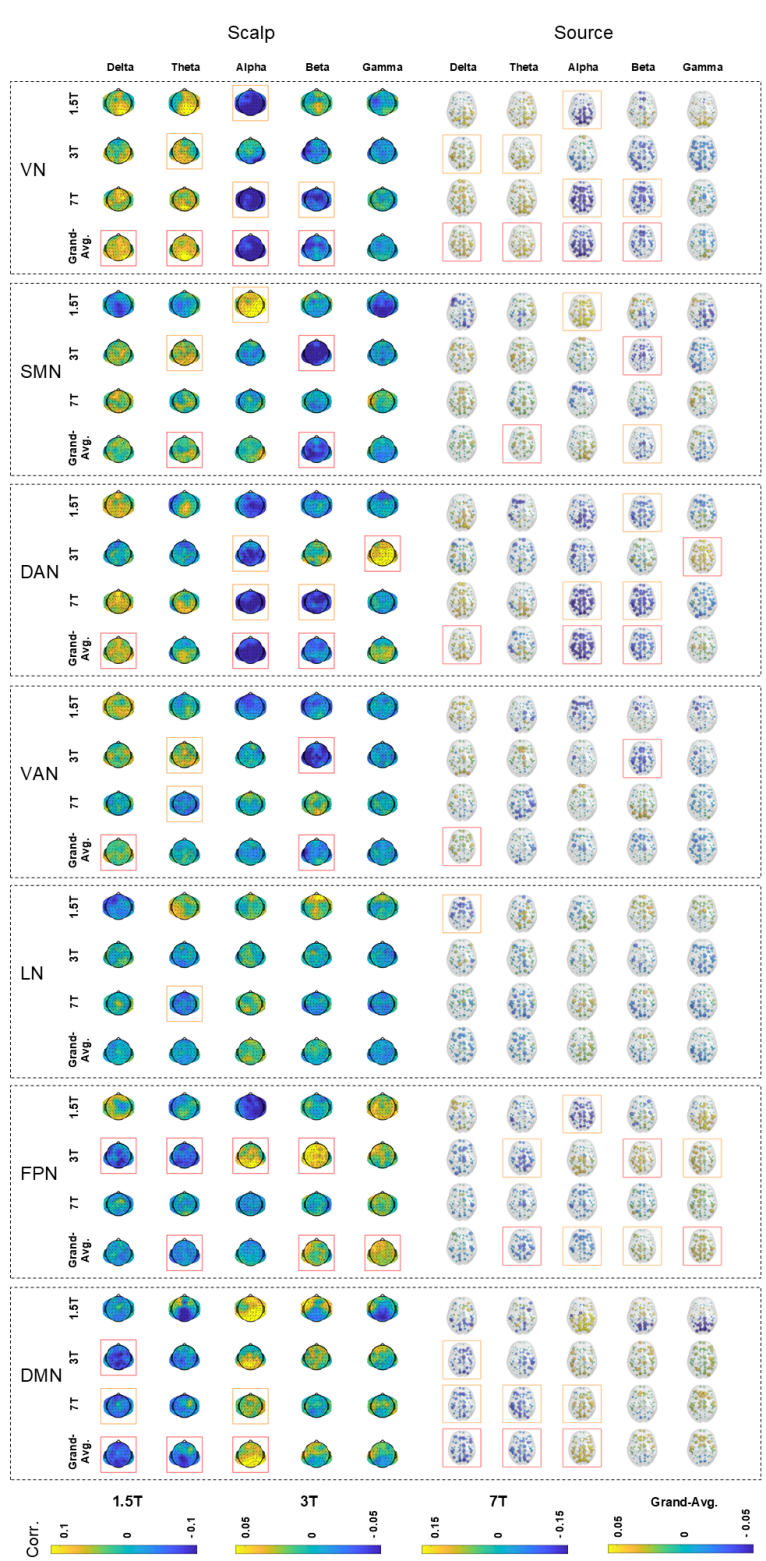
Spatial maps of EEG–fMRI temporal correlations with a 6-s HRF delay. The subject-averaged spatial maps of EEG–fMRI temporal correlations obtained using the canonical HRF with a 6-s overshoot delay are shown for each fMRI RSN (rows) and EEG frequency band (columns) in both scalp (channels) and source (regions of the Desikan atlas) spaces (left–right). Results are presented for each of the individual datasets (1.5T, 3T, and 7T) and for the grand average across datasets. Boxes highlight where the correlations are significantly different from zero (p < 0.05) based on one-sample t-tests across subjects: orange boxes indicate uncorrected results, and red boxes indicate that remain significant False Discovery Rate correction. The figure illustrates mostly spatially homogeneous correlation patterns, similar between scalp and source spaces. While some of these are dataset specific, there are several common trends across datasets, including positive δ and θ correlations, and negative α and β correlations, in the VN and DAN, as well as the opposite pattern (positive α/β and negative δ/θ correlations) in the DMN. Discrepancies across datasets are noted, such as opposite α correlations with the SMN at 1.5T versus 3T and 7T, and differing α and β correlations with the FPN between the 3T (eyes closed) and the 1.5T/7T (eyes open) datasets. Acronyms: RSN (resting-state network), HRF (hemodynamic response function), VN (visual network), SMN (somatomotor network), DAN (dorsal attention network), VAN (ventral attention network), LN (limbic network), FPN (frontoparietal network), DMN (default mode network), EC (eyes closed), EO (eyes open).

**Fig. 3.**
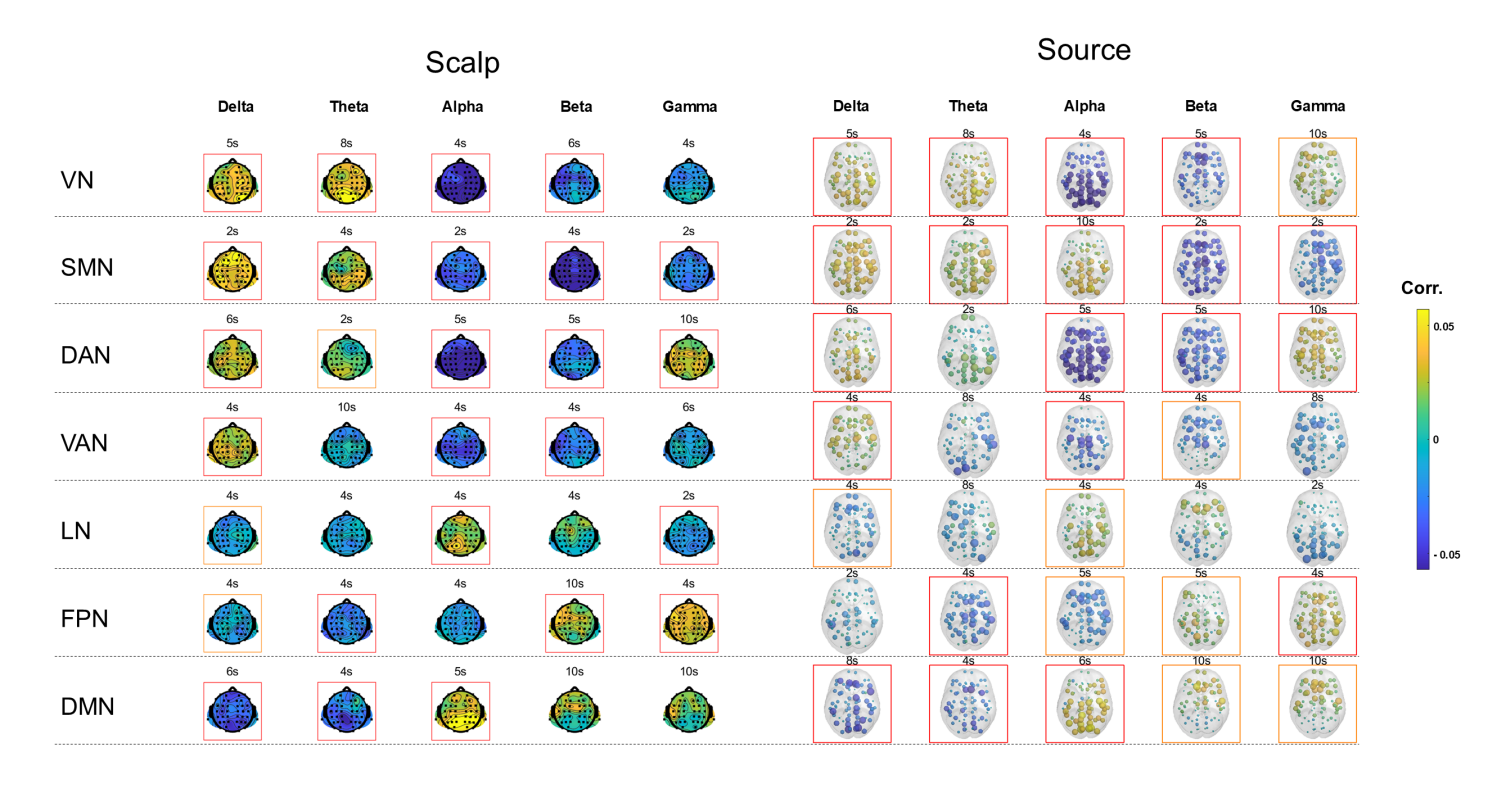
Spatial maps of the grand average EEG–fMRI temporal correlations for the best HRF delay. The grand average spatial maps of EEG–fMRI temporal correlations, averaged across all subjects from the 1.5T, 3T, and 7T datasets, are displayed for each RSN (rows) and EEG frequency band (columns) in both scalp (channels) and source (Desikan–Killiany atlas regions) spaces (left–right). For each combination of RSN, frequency band, and EEG space, the “best” HRF delay was selected as the one yielding the highest absolute spatially averaged correlation value. Boxes indicate spatially averaged correlations that are significantly different from zero (p < 0.05) based on one-sample t-tests across subjects: orange boxes mark uncorrected results, while red boxes mark those that survive False Discovery Rate correction. Acronyms: RSN (resting-state network), HRF (hemodynamic response function), VN (visual network), SMN (somatomotor network), DAN (dorsal attention network), VAN (ventral attention network), LN (limbic network), FPN (frontoparietal network), DMN (default mode network), EC (eyes closed), EO (eyes open).

**Fig. 4.**
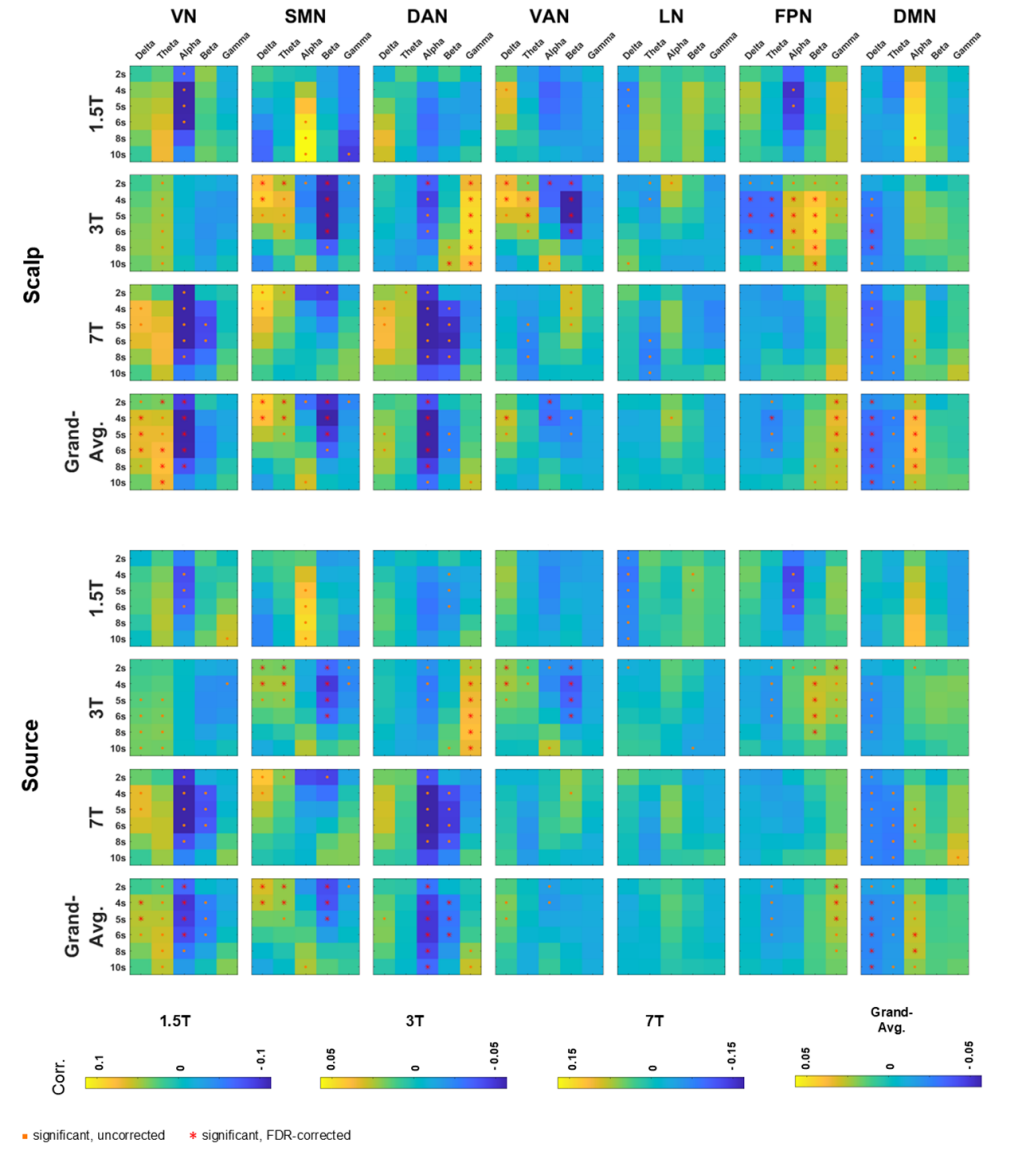
EEG–fMRI spatially averaged temporal correlations across HRF delays. The heatmaps show spatially averaged EEG–fMRI correlations, averaged across subjects, for each dataset (1.5T, 3T, and 7T), and for the grand average across all datasets, separately for scalp and source spaces. Each subplot corresponds to a particular dataset or the grand average and to one of the seven RSNs. Within each subplot, rows represent different HRF delays (2, 4, 5, 6, 8, and 10 s), and columns represent different EEG frequency bands (δ, θ, α, β, γ). Significant correlations, determined by one-sample t-tests against zero (p < 0.05), are indicated by orange dots for uncorrected results and red asterisks for False Discovery Rate-corrected results. Significant findings are observed for various combinations of RSNs, frequency bands, and HRF delays. The 3T dataset (EC) shows non-significant α–VN correlations for all HRF delays, contrasting with significant negative α–VN correlations for several HRF delays in the 1.5T and 7T datasets (EO). Overall, correlation values tend to change smoothly as the HRF delay increases, with some cases showing biphasic patterns. For example, across datasets, α–SMN correlations transition from negative or neutral values at shorter HRF delays to positive values at longer delays. These general patterns are qualitatively similar in both scalp and source EEG spaces. Acronyms: RSN (resting-state network), HRF (hemodynamic response function), VN (visual network), SMN (somatomotor network), DAN (dorsal attention network), VAN (ventral attention network), LN (limbic network), FPN (frontoparietal network), DMN (default mode network), EC (eyes closed), EO (eyes open).

**Fig. 5.**
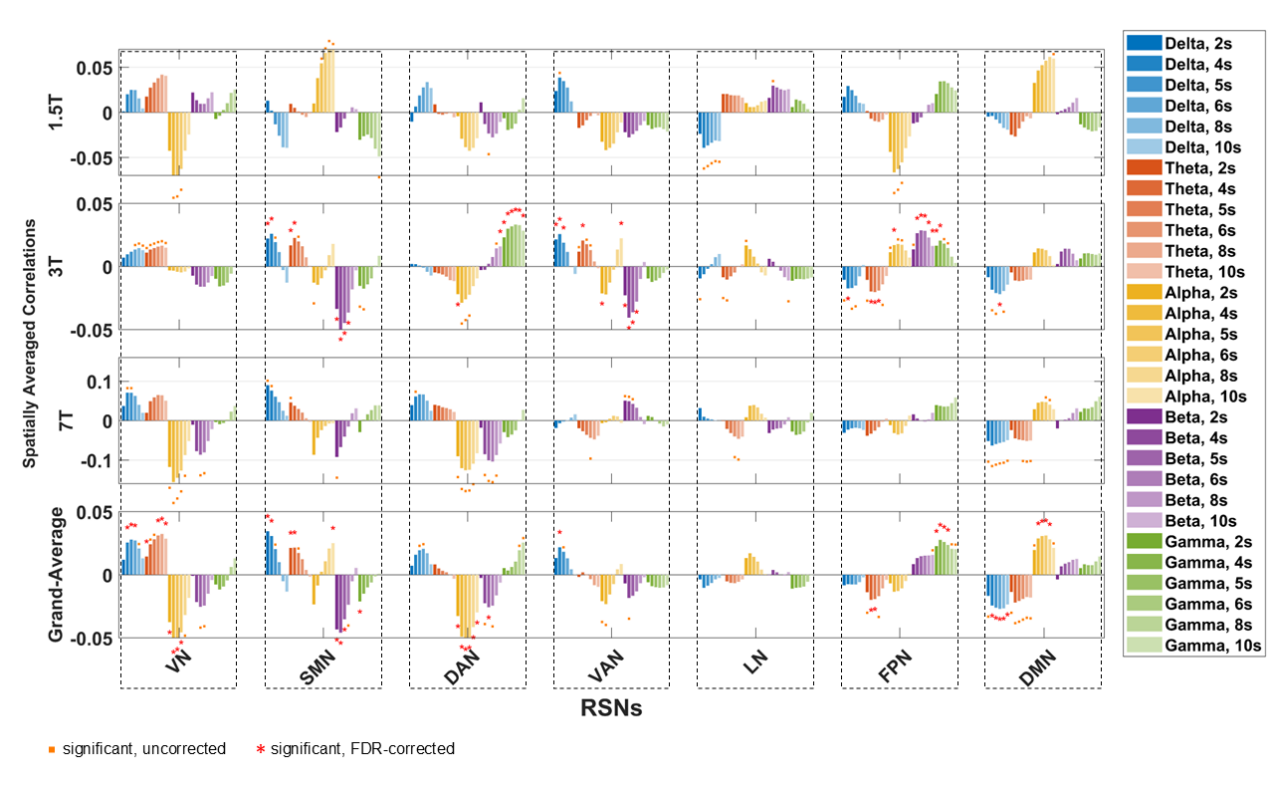
EEG–fMRI spatially averaged temporal correlations pooled across scalp and source EEG spaces. The bar plots show spatially averaged EEG–fMRI correlations, averaged across subjects, for each dataset (1.5T, 3T, and 7T), and for the grand average across all datasets, pooled across scalp and source EEG spaces. Results for the seven RSNs are displayed from left to right, for each EEG frequency band (δ, θ, α, β, γ; colors) and HRF delay (2, 4, 5, 6, 8, and 10 s; hues). Significance was determined using one-sample t-tests against zero (p < 0.05): orange dots mark uncorrected results, and red asterisks mark results that remain significant after False Discovery Rate correction. Acronyms: RSN (resting-state network), HRF (hemodynamic response function), VN (visual network), SMN (somatomotor network), DAN (dorsal attention network), VAN (ventral attention network), LN (limbic network), FPN (frontoparietal network), DMN (default mode network), EC (eyes closed), EO (eyes open).

Figure 2shows the spatial maps of EEG–fMRI temporal correlations for each fMRI RSN and EEG frequency band, derived in both scalp and source spaces, with the canonical HRF delay 6 s. The maps were averaged across subjects within each individual dataset (1.5T, 3T, and 7T), as well as across all datasets to generate a grand-average map.Supplementary Figures S4–S8present the corresponding results obtained with HRF delays of 2, 4, 5, 8, and 10 s, respectively. Distinct correlation patterns were identified between fMRI RSNs and EEG band power for specific HRF delays, revealing complex interactions between RSN, frequency band, and HRF delay.Figure 3shows correlation spatial maps for the “best” HRF delay associated with each combination of frequency band, RSN, EEG space, and dataset (including the grand average). The “best” delay was defined as the one yielding the highest spatially averaged absolute correlation value.Figure 4provides an overview of these results by displaying the spatially averaged temporal correlations for all HRF delays, from 2 to 10 s, illustrating how the overall correlation patterns continuously evolve as the EEG-fMRI delay varies.Figure 5presents a complementary view of these spatially averaged correlations, reorganized and pooled across scalp and source spaces.Supplementary Figure S9provides separate results for scalp- and source-space data.

#### 3.1.1 Consistency within subjects and across datasets (T-tests)

Each of the figures described above presents the results of the one-sample t-tests against zero conducted on the spatially averaged correlations. These tests were performed for each individual dataset (1.5T, 3T, and 7T) to assess consistency across subjects within that dataset, and for the grand average data to evaluate consistency across datasets. In each figure, we highlighted the spatial maps that achieved significance (p < 0.05), both before and after applying FDR correction for multiple comparisons.

#### 3.1.2 Interactions between fMRI RSNs, EEG frequency bands, and HRF delays (ANOVA)

The four-way repeated measures ANOVA revealed significant main effects for dataset (F = 7.7, p < 0.001), RSN (F = 5.0, p < 0.001), frequency band (F = 25.5, p < 0.001), and HRF delay (F = 3.1, p = 0.008). In terms of pairwise interactions, significant findings were observed between dataset and RSN (F = 5.3, p < 0.001), dataset and frequency band (F = 14.8, p < 0.001), RSN and frequency band (F = 39.2, p < 0.001), and frequency band and HRF delay (F = 2.5, p < 0.001). Additionally, a significant triple interaction was identified between dataset, RSN, and frequency band (F = 17.8, p < 0.001). No significant quadruple interactions were found.

##### 3.1.2.1 Interaction between dataset, RSN, and frequency band

Post hoc analyses, adjusted with Bonferroni correction for a family of 105 (3 datasets x 7 RSNs x 5 frequency bands), were conducted to explore the significant (p < 0.05) three-way interaction identified between dataset, RSN, and frequency band. Specifically, the following RSN frequency band pairs showed significant differences in correlations across datasets: alpha- and beta-VN; delta-, alpha-, and gamma-SMN; delta-, theta-, alpha-, beta-, and gamma-DAN; theta- and beta-VAN; theta-LN; alpha-FPN; alpha- and gamma-DMN.

##### 3.1.2.2 Interaction between frequency band and HRF delay

Post hoc analyses, adjusted with Bonferroni correction for a family of 30 (5 frequency bands × 6 HRF delays), were conducted to explore the significant (p < 0.05) two-way interaction identified between frequency bands and HRF delays. Specifically, for the 2-s delay, correlations in both the delta and theta bands were significantly higher than in the alpha band. Similar patterns were noted for the 4- and 5-s delays, where delta and theta bands consistently exhibited higher correlations than the alpha and beta bands. At the 6-s delay, the delta band again yielded significantly higher correlations than the alpha band. No significant differences were identified within each band across the various delays.

### 3.2 Complementary analyses

#### 3.2.1 Effect of the number of subjects and scan duration

**Fig. 6.**
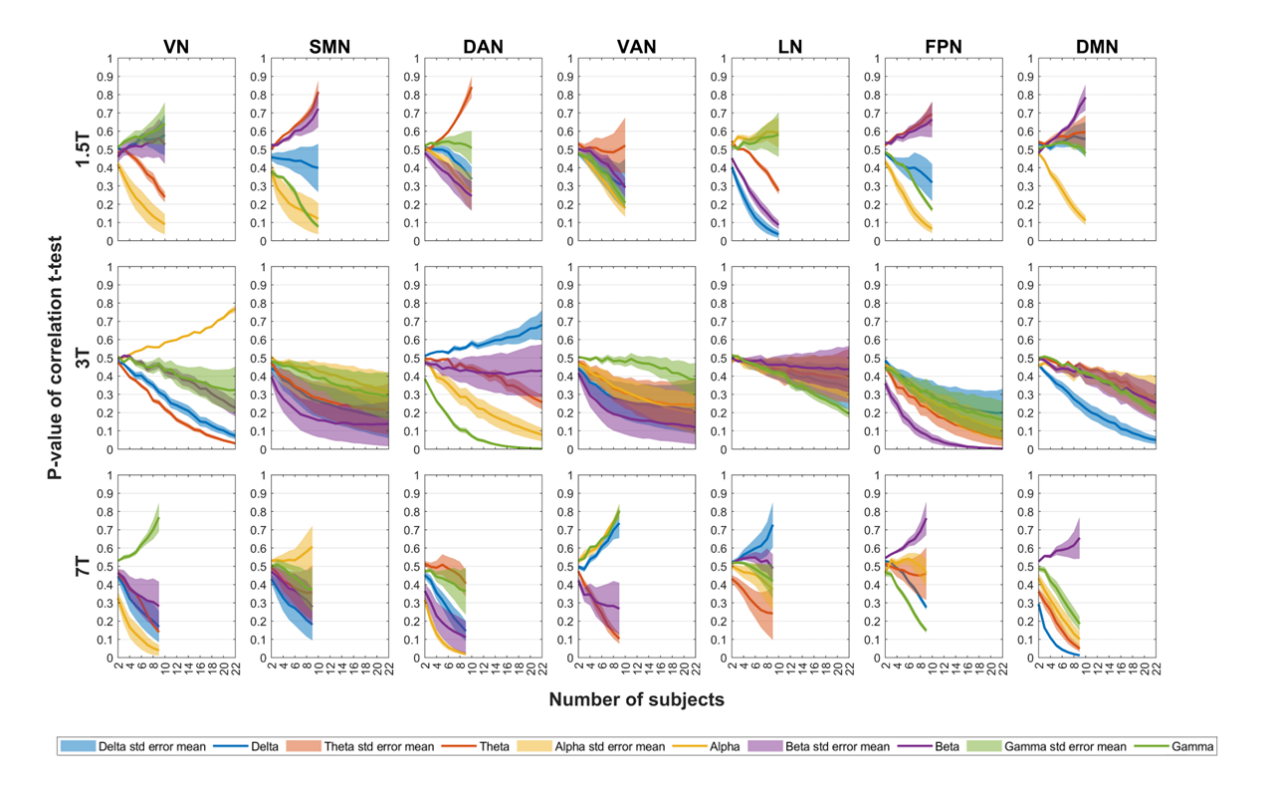
Effect of the number of subjects on the significance of EEG–fMRI correlations. Impact of increasing number of subjects on the p-value of the t-statistics derived from EEG-fMRI spatially averaged temporal correlations. Data were pooled across scalp and source EEG spaces to derive average correlation values. Distinct colors denote EEG band power across δ, θ, α, β, and γ frequency bands, with shaded areas indicating the standard mean error across a set of HRF delays (2, 4, 5, 6, 8, and 10 s). Rows correspond to each EEG-fMRI dataset (1.5T, 3T, and 7T), while columns correspond to the seven canonical fMRI RSNs. For each dataset, subjects were randomly sampled (ranging from 1 to n subjects, over 5000 iterations) prior to computing the t-stat values. Acronyms: RSN (resting-state network), HRF (hemodynamic response function), VN (visual network), SMN (somatomotor network), DAN (dorsal attention network), VAN (ventral attention network), LN (limbic network), FPN (frontoparietal network), DMN (default mode network), EC (eyes closed), EO (eyes open).

Figures 6and7present the effects of varying the sample size and the scan duration on the significance of EEG–fMRI temporal correlations, respectively.Supplementary Figures S10–S13present these effects separately for the scalp and source spaces, as well as the effect of these factors in the correlations themselves.

**Fig. 7.**
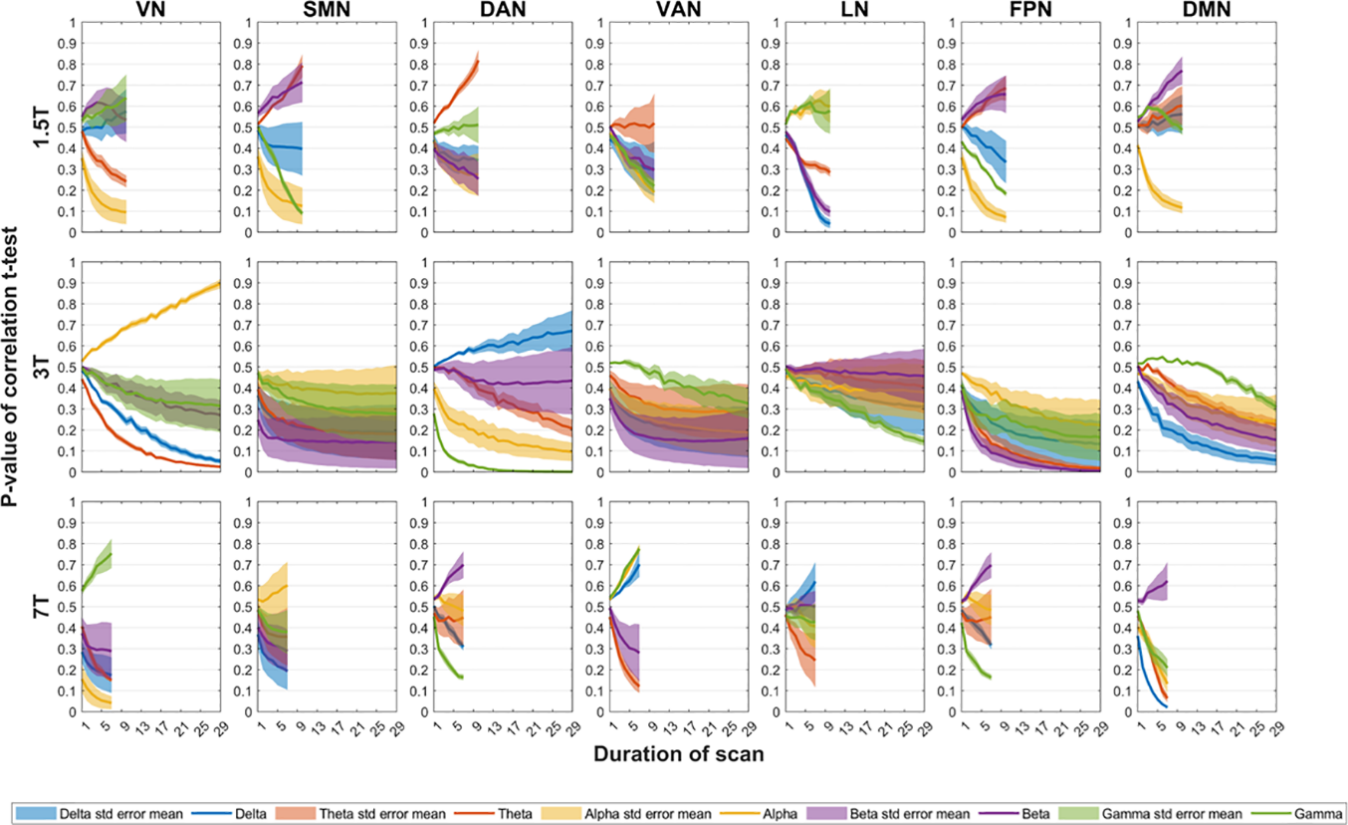
Effect of the scan duration on the significance of EEG–fMRI correlations. Impact of increasing scan duration on the p-value of the t-statistics derived from EEG-fMRI spatially averaged temporal correlations. Data were pooled across scalp and source EEG spaces to derive average correlation values. Distinct colors denote EEG band power across δ, θ, α, β, and γ frequency bands, with shaded areas indicating the standard mean error across a set of HRF delays (2, 4, 5, 6, 8, and 10 s). Rows correspond to each EEG-fMRI dataset (1.5T, 3T, and 7T), while columns correspond to the seven canonical fMRI RSNs. For each dataset, segments of data were randomly selected (ranging from 1 to n consecutive minutes, over 5000 iterations) prior to computing the temporal correlations and t-stat values. Acronyms: RSN (resting-state network), HRF (hemodynamic response function), VN (visual network), SMN (somatomotor network), DAN (dorsal attention network), VAN (ventral attention network), LN (limbic network), FPN (frontoparietal network), DMN (default mode network), EC (eyes closed), EO (eyes open).

#### 3.2.2 Correlations across extended HRF delays

**Fig. 8.**
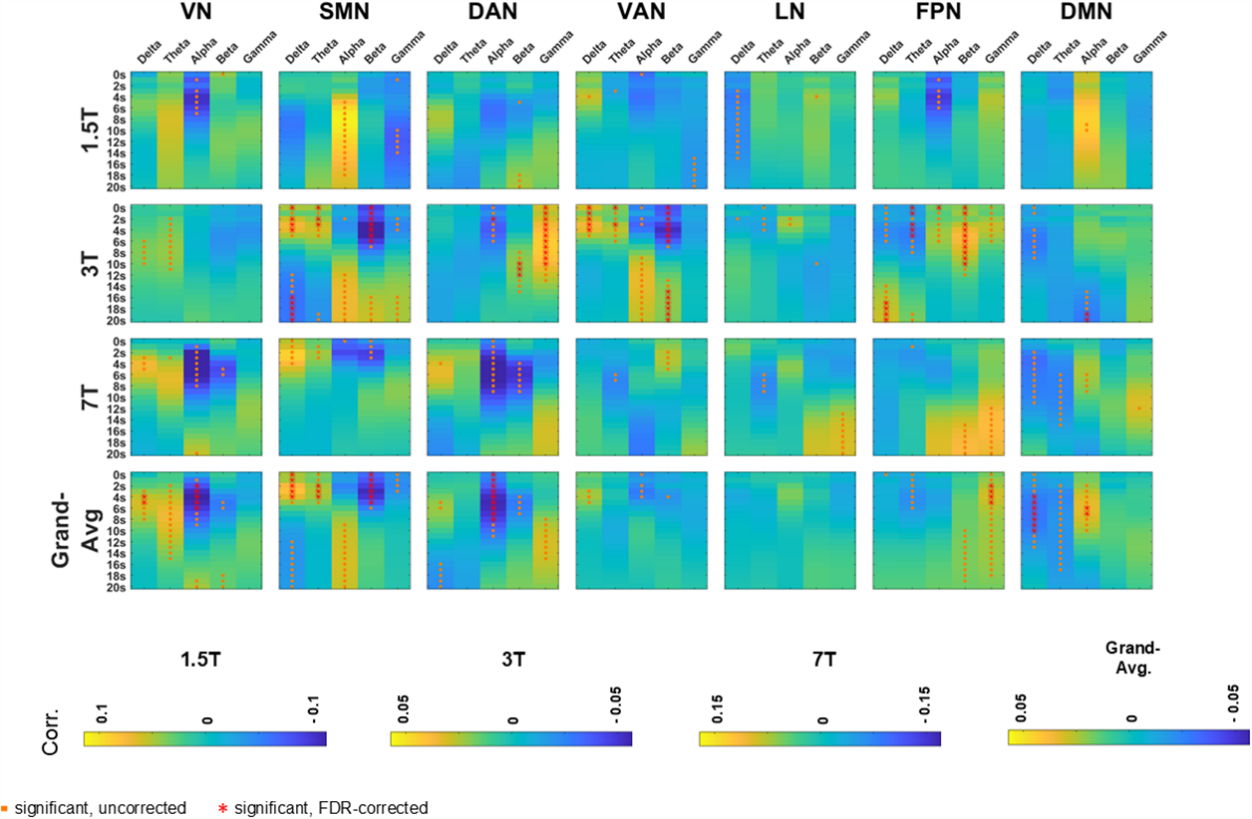
EEG–fMRI spatially averaged temporal correlations across an extended range of HRF delays. The heatmaps show spatially averaged EEG–fMRI correlations, averaged across subjects, for each dataset (1.5T, 3T, and 7T), and for the grand average across all datasets, pooled across scalp and source spaces. Each subplot corresponds to a particular dataset or the grand average and to one of the seven RSNs. Within each subplot, rows represent different HRF delays (ranging from 0 to 20 s), and columns represent different EEG frequency bands (δ, θ, α, β, γ). Significant correlations, determined by one-sample t-tests against zero (p < 0.05), are indicated by orange dots for uncorrected results and red asterisks for False Discovery Rate-corrected results. Significant findings are observed for HRF delays both shorter and longer than those commonly considered in studies (2–10 s). Acronyms: RSN (resting-state network), HRF (hemodynamic response function), VN (visual network), SMN (somatomotor network), DAN (dorsal attention network), VAN (ventral attention network), LN (limbic network), FPN (frontoparietal network), DMN (default mode network), EC (eyes closed), EO (eyes open).

Figure 8explores EEG–fMRI correlations and their significance, across an extended range of HRF overshoot delays, from 0 to 20 s.Supplementary Figure S14provides separate results for scalp- and source-space data.

## 4 Discussion

By systematically analyzing three independent resting-state simultaneous EEG-fMRI datasets, we found consistent temporal correlations, across subjects and datasets, between the seven canonical RSNs and the concurrent EEG band power in five canonical frequency bands. Each RSN exhibited a distinct, frequency- and delay-dependent spatial distribution of correlation with EEG power co-fluctuations. Each spatial distribution exhibited a main polarity (either positive or negative), with no evidence of opposite polarities within the same map, which prompted the use of spatially averaged correlations in subsequent statistical analyses, simplifying interpretation via dimensionality reduction. Significant variations were observed in the spatially averaged EEG–fMRI RSN correlations across different RSNs and EEG frequency bands, which also significantly varied with the HRF delays considered. Additionally, since no significant main effects or interactions were found for the EEG space (scalp vs. source) on EEG–fMRI RSN correlations, a pooling strategy was adopted in several analyses, averaging both scalp- and source-space spatially averaged correlations together.

### 4.1 Relation with previous studies

Below we discuss how our findings align with (or diverge from) previous EEG-fMRI studies that have investigated the relationship between fMRI RSNs and EEG band power fluctuations. Our principal goal is to interpret the consistency of the observed EEG–fMRI correlation patterns, both across subjects and datasets, focusing on each RSN separately.

#### 4.1.1 Visual network

Across datasets, the fMRI VN showed significant positive correlations with delta/theta EEG power and significant negative correlations with alpha/beta bands for a range of HRF delays (primarily between 2 and 8 s), consistent with previous studies (Jann et al., 2010;Mantini et al., 2007;Meyer et al., 2013). In the eyes-open datasets (1.5T and 7T), we observed significant (uncorrected) negative alpha–VN correlations across subjects, while the eyes-closed 3T dataset did not yield a significant alpha-VN effect. This is surprising, given the well-established role of alpha oscillations in sensory inhibition, in particular in visual processing, as exemplified by the Berger effect. Indeed, numerous studies have reported negative co-fluctuations between occipital alpha power and fMRI VN activity in both EO and EC resting state (Feige et al., 2005;Goldman et al., 2002;Jann et al., 2010;Laufs et al., 2006;Mantini et al., 2007;Meyer et al., 2013;Moosmann et al., 2003).

In our 3T dataset (EC), while negative alpha–VN correlations were present in occipital regions, they did not reach significance once averaged across all channels or source-space regions. This finding is less surprising when considering previous work showing that, under EC conditions, alpha-driven suppression may be localized primarily to posterior regions, with other cortical areas displaying near-zero or even positive correlations (Jann et al., 2010). Such spatial heterogeneity, clearly observed in our 3T EC dataset (Fig. 2), likely diminished the significance of the spatially averaged alpha–VN correlations. In contrast, the EO datasets (1.5T and 7T) showed more spatially uniform negative alpha–VN correlations, resulting in significant spatially averaged effects.

#### 4.1.2 Somatomotor network

Across datasets, the fMRI SMN showed significant positive correlations with delta/theta power (primarily between 2 and 4 s HRF delays), along with significant negative correlations with beta power (~2–5 s HRF delays), in line with previous findings (Jann et al., 2010;Mantini et al., 2007;Meyer et al., 2013). Given that theta rhythms are often associated with sensorimotor integration (Caplan et al., 2003), the positive correlations observed might reflect coordinated neural activity within the SMN during rest. In contrast, the beta central rhythm has been traditionally linked to motor control and tends to decrease during movement (Pfurtscheller, 1981). Hence, the negative correlations observed suggest an inverse relationship between motor readiness and SMN BOLD activity. In fact, both beta and alpha synchronizations have been described as correlates of “idling” motor function, being inversely correlated with BOLD activity in the somatosensory and motor cortices (Ritter et al., 2009). It has been shown that alpha band sensorimotor rhythm (SMR), or mu rhythm, is negatively correlated with the SMN during resting state (Tsuchimoto et al., 2017;Yin et al., 2016). However, the correlation between the alpha band and the SMN varied substantially across datasets. While the 3T and 7T datasets generally showed the expected negative correlations (although often not reaching significance) between alpha and the SMN (Jann et al., 2010;Meyer et al., 2013), the 1.5T dataset instead revealed significant (although uncorrected) positive alpha–SMN correlations (~6–10 s HRF delays).DiFrancesco et al. (2008)reported positive correlations between occipital alpha power and SMN activity, suggesting that different alpha rhythms (e.g., occipital vs. central or μ rhythm) may relate differently to SMN BOLD signals. However, this does not fully explain the 1.5T results, since even central alpha power exhibited positive correlations with the SMN in that dataset (seeFig. 2). A deeper analysis into the dynamics of these correlations with varying HRF delays provides additional insight into these discrepancies. In all datasets studied, the correlation between alpha power and the SMN evolves from near-zero or negative values at shorter HRF delays to more positive or near-zero values at longer HRF delays, indicating a biphasic hemodynamic relationship, as observed elsewhere (Prokopiou et al., 2022). In the 1.5T dataset, this transition to positive correlations occurs around a 6-s delay, while in the 3T and 7T datasets, it emerges later, around 10–12 s. By extending the range of HRF delays (as shown inFig. 8), this biphasic pattern becomes evident and even yields significant (uncorrected) positive correlations across datasets for HRF delays greater than 10 s.

#### 4.1.3 Dorsal attention network

Across datasets, the DAN showed significant (uncorrected) positive correlations with the delta power at 5–6 s HRF delays, negative correlations with the alpha and beta bands that were strongest at around 4–6 s delays, and positive correlations with the gamma band from 8 s onward. These alpha and beta results are consistent with previous studies that have explicitly reported co-fluctuations between the fMRI DAN and EEG band power (Mantini et al., 2007;Sadaghiani et al., 2010). Additionally, two studies (Laufs et al., 2003b,^2006^) reported significant negative co-fluctuations between the alpha power and the fMRI signal in the superior parietal cortex, a region within the DAN.

#### 4.1.4 Ventral attention network

In the VAN, both the 1.5T and 3T datasets exhibited significant positive correlations with the delta band. Additionally, the 3T dataset showed significant positive correlations with the theta band, whereas the 7T dataset displayed significant negative correlations. Although these patterns were not always significant within each individual dataset, all three datasets consistently showed negative alpha correlations at shorter HRF delays, resulting in a significant negative alpha–VAN correlation across datasets for HRF delays of 2–4 s. While no previous studies have directly examined relationships between the fMRI VAN and EEG band power, several have reported negative co-fluctuations between alpha power and BOLD activity in the inferior frontal cortex, a region within the VAN (Laufs et al., 2003a,^2006^;Moosmann et al., 2003;Scheeringa et al., 2008). Negative beta–VAN correlations were found for the 3T dataset, whereas the 7T dataset showed positive correlations.Laufs et al. (2003b)also reported that beta power in the temporoparietal junction (part of the VAN) may correlate positively or negatively with BOLD activity depending on the beta frequency subrange. Specifically, lower beta frequencies (17–23 Hz) showed positive correlations, while higher beta frequencies (23–30 Hz) showed negative correlations. This frequency-specific effect may help explain the divergent beta–VAN correlation patterns observed in our 3T and 7T datasets.

#### 4.1.5 Limbic network

In some datasets, the LN displayed significant (though uncorrected) negative co-fluctuations with the delta and theta bands, and positive correlations with the alpha and beta bands. However, when considering all datasets, these correlations generally did not reach significance. While no studies explicitly report relationships between the fMRI LN and EEG band power, some studies reported positive co-fluctuations between the alpha EEG power and the fMRI signal in regions belonging to the limbic network: the insular cortex (Goldman et al., 2002) and the anterior cingulate cortex (ACC;DiFrancesco et al., 2008).

#### 4.1.6 Frontoparietal network

Across datasets, the FPN yielded significant positive correlations with the gamma power, at around 2–6 s HRF delays. In the 3T dataset, the FPN showed significant negative co-fluctuations with delta/theta power, as well as positive co-fluctuations with beta/gamma power, over a wide range of HRF delays, consistent with previous studies (Jann et al., 2010;Mantini et al., 2007). Alpha power co-fluctuations varied substantially across the three datasets, being significantly positive for the 3T dataset, but negative for the 1.5T and 7T datasets. Interestingly, an eyes-open resting-state study byMeyer et al. (2013)reported negative alpha–FPN correlations, as well as positive theta–FPN and negative beta–FPN correlations, mirroring the patterns found in our EO datasets (1.5T and 7T) and contrasting with the EC 3T dataset. This raises the question of whether disparities found between datasets could be influenced by the EO versus EC condition. Notably, the eyes-closed study byJann et al. (2010)found both positive and negative alpha correlations with the FPN, depending on the scalp region considered: positive alpha-1 (8.2–10.5 Hz) and alpha-2 (10.5–14.0 Hz) in occipital electrodes and negative α-2 in frontal electrodes. This spatial pattern closely resembles that found in our 3T EC dataset (seeFig. 2), where the positive alpha correlations were primarily localized to occipital channels.

#### 4.1.7 Default mode network

Across datasets, the DMN showed significant negative co-fluctuations with the delta/theta power for a wide range of delays (2–10 s) and positive co-fluctuations with the alpha band, primarily at HRF delays around 4–8 s, aligning with previous studies (Jann et al., 2010;Mantini et al., 2007). Also consistent with our observations,Scheeringa et al. (2008)identified correlations between theta power and DMN regions, such as the medial prefrontal cortex (mPFC), inferior parietal cortex, and anterior cingulate cortex (ACC). However, alpha–DMN relationships remain a matter of discordance in much of the existing literature, with both negative and positive correlations having been reported. For example,Mo et al. (2013)found positive alpha–DMN correlations in eyes-open rest but not in eyes-closed rest, contrasting with other studies reporting negative alpha–DMN correlations (Meyer et al., 2013) or negative alpha correlations with DMN regions such as the ACC and inferior parietal cortex (Goldman et al., 2002;Laufs et al., 2003a,^2006^;Moosmann et al., 2003). In contrast,DiFrancesco et al. (2008)documented positive alpha–ACC co-fluctuations.Bowman et al. (2017)further highlighted this complexity by identifying both positive and negative alpha–DMN relationships, depending on the specific DMN sub-network considered, suggesting that the DMN may simultaneously support both introspective and externally oriented processes. Similarly,Marawar et al. (2017)also reported both positive and negative EEG–BOLD correlations in different DMN regions for delta and theta bands. These more recent studies highlight the complexity of the frequency modulation of the DMN, potentially explaining the varied and seemingly contradictory findings in the literature. Nevertheless, our data converged on a pattern of negative delta–/theta–DMN and positive alpha–DMN correlations.

#### 4.1.8 Interactions between RSNs

The DAN and DMN demonstrated generally inverse co-fluctuations across the delta, theta, alpha, and beta bands, potentially reflecting the well-documented anti-correlation between the activity of task-positive and task-negative networks in both task and rest conditions (Chang et al., 2013;Fox et al., 2005). Traditionally, the DMN has been correlated with activity during rest and internally oriented tasks, while the DAN has been associated with attention-demanding and externally oriented tasks. This anti-correlation might represent a modulation in the frequency domain of brain networks, potentially competing for neural resources (Mantini et al., 2007). In alignment with this, theta power, often linked with sustained attention, exhibits positive co-fluctuations with the DAN and negative co-fluctuations with the DMN, reflecting its role in managing internal and external attention. Conversely, alpha power, which has been associated with the suppression of attention to the external environment, demonstrates negative co-fluctuations with the DAN and positive co-fluctuations with the DMN (Magosso et al., 2021).

The divergences observed in FPN co-fluctuation patterns across the 3T, 1.5T, and 7T datasets might be interpreted under a related argument. The FPN is traditionally linked to executive control and decision making (Vincent et al., 2008), which may either relate to perceptual (externally oriented) or introspective (internally oriented) cognitive processes. The literature suggests that the FPN is functionally connected to both the DMN and the DAN (Spreng et al., 2013) and recent studies further explored this notion, investigating functional heterogeneity within the FPN (Braga & Buckner, 2017;Dixon et al., 2018).Dixon et al. (2018)identified two main subsystems within the FPN, FPN-A, and FPN-B, evident in task performance and resting state, with distinct roles in executive control. The former, functionally connected to the DMN, was theorized to be activated during internally directed attention. The latter, functionally connected to the DAN, was proposed to participate mainly in perceptual attention, facilitating interactions with the environment. In this context, our findings suggest that the FPN configuration in the 3T dataset might predominantly reflect the characteristics of the FPN-A subsystem, presenting mostly positive alpha co-fluctuations and negative theta co-fluctuations, similarly to the DMN. In contrast, the FPNs in the 1.5T and 7T datasets seem to align with the FPN-B subsystem, showing mostly negative alpha co-fluctuations, similarly to the DAN. An additional hypothesis considers the role of eyes-open versus eyes-closed condition in potentially modulating the dominant FPN mode. The eyes-open condition might promote a state of latent external attention, thereby possibly enhancing perceptual cognition. This condition could influence alpha co-fluctuations with the FPN, reflecting a subtle continuous engagement with the external stimuli, consistent with results from the eyes-open datasets.

However, both the limbic network and the DMN exhibited often similar EEG–fMRI correlation patterns in the delta, theta, alpha, and beta bands, which might be reflective of their related roles in internal cognition, emotional processing, and memory recall during resting state (Greicius et al., 2003;Stephani, 2014). Indeed, these two networks also commonly share overlapping brain regions such as the ACC and the mPFC.

Finally, given the external attention orientation of both the DAN and VAN (Fox et al., 2006), a cooperative modulation of these two networks, leading to similar co-fluctuations with the same frequency bands, is a logical expectation and was partially observed in our results.

#### 4.1.9 Interactions between frequency bands

Another noteworthy observation across datasets is that RSN correlations with the delta and theta bands often exhibit patterns that are inverse of those observed with the alpha and beta bands. This may reflect distinct neural mechanisms or cognitive states associated with both pairs of frequency bands. Specifically, cross-frequency synchronization (CFS) between alpha and beta rhythms has been implicated in attentional processes, especially during rest, where it may facilitate information integration across brain networks (Nikulin & Brismar, 2006;Sacchet et al., 2015).Palva and Palva (2018)further proposed that synchrony among different frequency bands at rest contributes to the coordination of neural processing across distributed brain regions. Such mechanisms could underlie the inverse relationships we observe between delta/theta and alpha/beta RSN co-fluctuations. However, it is important to acknowledge that delta and theta bands can be particularly susceptible to contamination from residual BCG and motion artifacts (Debener et al., 2008;Fellner et al., 2016). Although our data were corrected for motion and pulse artifacts, and visual inspection confirmed their effective removal, some residual effects may persist in these bands, potentially influencing their co-fluctuations.

### 4.2 Impact of the hemodynamic delay

We found that the HRF delay at which significant EEG–fMRI correlations emerged varied depending on the RSN, frequency band, and dataset. Notably, the strongest correlations did not consistently occur at the canonical 6-s HRF delay commonly assumed in studies of EEG–fMRI RSNs (seeFig. 3). This variability may reflect the well-documented heterogeneity in neurovascular coupling across different brain regions and conditions (Logothetis & Wandell, 2004). These findings emphasize the importance of acknowledging variability in optimal HRF delays and having caution when interpreting or comparing EEG–fMRI correlation results derived from a single, canonical delay. They also highlight the importance of exploring a broad range of HRF delays, specifically from 2 to 10 s, to fully capture the temporal dynamics of EEG–fMRI co-fluctuations.

Interestingly, we also observed significant correlations at very short HRF delays, including overshoot delays of 0–2 s. For example, alpha–VN anticorrelations emerged at a 2-s delay, and beta–SMN and alpha–DAN anticorrelations at a 0-s delay (seeFig. 8). These results imply that the hemodynamic response may begin before the corresponding EEG response (seeSupplementary Fig. S3). Similar findings have been reported in epilepsy research, where BOLD changes can precede observable EEG discharges, presumably reflecting metabolically demanding neuronal activity that has not yet synchronized sufficiently to be detected by EEG (Hawco et al., 2007;Rollings et al., 2016). Although our study focused on healthy individuals, these results raise the possibility that early, pre-EEG hemodynamic events may also occur in the healthy brain at rest.

### 4.3 Impact of EEG space

The similarity in EEG–fMRI correlations between scalp- and source-space data, specifically the lack of significant interactions between this variable and HRF delays, suggests that the neurovascular coupling underlying the observed correlations is represented similarly in both spaces regardless of their difference in spatial specificity. Crucially, this observation could inform future research of EEG–fMRI correlations, by suggesting that, under specific conditions, using scalp data may provide results comparable with those derived from source-localized data, thereby offering a methodological simplification.

### 4.4 Impact of sample size

T-tests against zero were used to evaluate the significance of spatially averaged correlations across subjects, providing an assessment of consistency across subjects in correlation values for each dataset. Notably, only correlations from the 3T dataset remained significant following FDR correction. Given that this dataset comprised a considerably larger sample size (23 subjects) compared with the 1.5T and 7T datasets (10 and 9 subjects, respectively), a question arose regarding the sufficiency of the number of subjects (i.e., number of observations) to achieve statistically significant results across each dataset. By performing permutation-based tests, we found that the correlation p-values in general stabilized only when considering 8–12 subjects, which could be observed only in the case of the 3T dataset (seeFig. 6andSupplementary Fig. S11). This finding suggests that discrepancies in the significance of the EEG–fMRI correlations between the 3T dataset and the other two smaller datasets may arise from this sensitivity to the sample size (the number of subjects).

### 4.5 Impact of scan duration

The influence of scan duration on the correlations and their respective p-values was similarly explored, given the substantial discrepancies in scan durations among the three datasets (10 min for the 1.5T dataset, 30 min for the 3T dataset, and 8 min for the 7T dataset). The hypothesis is that correlations would stabilize and become more robust with more prolonged scan durations, potentially yielding reduced variability across subjects. This hypothesis was supported by the observation that correlations for the 3T dataset stabilize around a scan duration of 10–15 min (seeSupplementary Fig. S12), which surpasses the scan durations of the other two datasets. This stabilization is mirrored as well for the correlation p-values, which, in most instances, also stabilize around those scan durations (seeFig. 7andSupplementary Fig. S13). Such effects could be attributed to the higher correlation magnitudes observed for these durations, or alternatively, to the joint effect of higher magnitudes and greater consistency between subjects.

### 4.6 Limitations and future work

Our study has made significant progress in reconciling and elucidating some of the inconsistencies observed in the literature regarding the relationship between EEG band power and fMRI-derived RSN activity. However, several challenges remain. One limitation concerns the definition and identification of the RSNs themselves. As emphasized in the recent review byUddin et al. (2023), there is no standardized approach for defining or identifying RSNs, leading to variable results that are likely one of the main sources of inconsistency in studies exploring the relationship between EEG and fMRI RSNs.

Methods for defining RSNs vary widely and significantly impact their spatial maps. Networks can be delineated using predefined parcellations or data-driven methods such as spatial ICA or clustering, and the parameters guiding these data-driven approaches are often reported inconsistently. Additionally, there is no universal consensus on which regions constitute each network, nor on the nomenclature used, especially for networks beyond the widely recognized visual, somatomotor, and default mode networks. Model order selection in ICA further complicates matters, as higher model orders can decompose networks into multiple sub-networks. In our study, we attempted to mitigate these challenges by using a consistent number of ICA components across datasets. Nonetheless, variability in the spatial maps of the identified networks persisted across datasets (seeSupplementary Fig. S1), potentially influencing the observed differences in EEG–fMRI correlations. Although we did not systematically analyze this aspect, future research should investigate how variability in fMRI network spatial maps across datasets affects the consistency of EEG–fMRI correlations.

Another challenge concerns interindividual differences in network structure, highlighting the importance of capturing both common and subject-specific network characteristics. In this study, we employed group spatial ICA followed by dual regression, an approach that integrates group-level network estimates with individual variations. Even so, this variability could influence the consistency of network identification across individuals and, consequently, the magnitude of the subject-averaged EEG–fMRI correlations. Therefore, future research could consider how within-subject stability in network spatial maps relates to the consistency of the corresponding EEG–fMRI correlations.

Variations in arousal present an additional source of variability. Indeed, arousal levels are known to vary both within and between subjects during resting-state acquisitions, influencing the temporal dynamics of the EEG spectrum and fMRI RSNs (Joliot et al., 2024;Makeig & Jung, 1995;Tagliazucchi & Laufs, 2014). These variations can be particularly pronounced in longer acquisitions, such as the 3T dataset (30 min in total, although recorded in separate 10-min runs, which should mitigate this effect), where transitions into drowsiness or sleep are more likely. Additionally, the eyes-open versus eyes-closed conditions across datasets may strongly influence arousal states, with eyes-closed rest being typically associated with reduced alertness and a higher likelihood of drowsiness and sleep. This is especially true when compared with eyes-open conditions with visual fixation (Tagliazucchi & Laufs, 2014), as in the eyes-open datasets used in this study. Compared with wakefulness, different stages of sleep are associated with distinct EEG patterns, such as increased delta and theta activity, decreased alpha power, and the emergence of spindles and K-complexes during light sleep (Olbrich et al., 2009). Indeed, the alpha rhythm is known to represent distinct phenomena with different functional roles and spatial patterns during wakefulness and during sleep. Such phenomena are likely accompanied by changes in neurovascular coupling mechanisms, affecting the relationship between EEG and the BOLD signal depending on the sleep stage or level of arousal (Olbrich et al., 2009). This contributes to the broader challenge of understanding how different brain states during rest interact with EEG frequency bands, modulating their relationships with simultaneous fMRI (Kung et al., 2024). Monitoring and adjusting for varying arousal levels could help control for some of these effects in resting-state studies (Falahpour et al., 2018;Liu & Falahpour, 2020). Similarly, head motion can correlate with the power of EEG signals and contribute to variability between subjects and sessions. Specifically,Fellner et al. (2016)reported that motion created spurious correlations with EEG signals below 20 Hz.

It is also important to acknowledge that functional connectivity is not a static feature but fluctuates across multiple temporal scales, even within the duration of a single scan session (Chang & Glover, 2010). Addressing this aspect requires methodologies that can capture how these time-varying dynamics contribute to the evolving patterns of the connectome over time (Keilholz et al., 2017). Additionally, understanding the implications of these dynamic changes for the correlations between EEG power and fMRI signals is crucial. Future studies could also consider the temporal evolution of these correlations, focusing on dynamic rather than static correlations to capture their time-varying relationship.

Another important limitation relates to the variability in data quality across datasets, driven mostly by differences in acquisition setups. For example, in the 1.5T dataset, the fMRI data have a reduced signal-to-noise ratio and spatial resolution compared with the 3T and 7T datasets. Meanwhile, the 7T has a significantly shorter TR (1.0 s) than the 1.5T and 3T datasets (2.6 and 2.0 s, respectively), providing finer temporal resolution. However, despite the superior fMRI data quality with higher field strengths, the EEG data quality tends to be poorer due to more severe MR-induced artifacts (e.g.,Jorge et al., 2015). Finally, we would like to note that for all datasets, we retained the preprocessing steps established in the previous studies by the collaborators of this study, as described in the respective papers (Deligianni et al., 2014;Jorge et al., 2019;Sadaghiani et al., 2010). While most preprocessing steps were consistent across datasets, some differences reflect the pipelines originally used in those studies. To improve comparability, the subsequent processing steps specific to this study were standardized across datasets. However, we believe that the disparities in acquisition setups and data preprocessing further contribute to the methodological heterogeneity that we wished to address. Our results, therefore, reflect the consistency in EEG–fMRI correlations that can be achieved despite methodological differences in data acquisition and preprocessing.

## 5 Conclusions

Faced with the extensive literature on EEG–fMRI correlations, in particular relating EEG band-specific power to fMRI RSNs, summarizing the varied and sometimes contradictory conclusions proves challenging due to numerous methodological variations. These span from dataset discrepancies, which inherently influence results due to variations in acquisition setups and study design, to differing approaches in data preprocessing and analysis methods that combine the two modalities. Our study systematically examined EEG–fMRI correlations, taking into account key parameters such as HRF delay and the space of EEG data (scalp or source space), while evaluating their consistency across subjects and generalization across different datasets. These datasets varied in fMRI field strength, number of participants, scan duration, and resting-state conditions (eyes open vs. eyes closed). Moreover, our systematic analysis carefully explored the spatial distribution of correlation values in both EEG scalp and source spaces. Through this approach, we not only found EEG–fMRI RSN correlations that were consistent across datasets, but also highlighted the impact of the explored factors, providing a clear perspective to understand some of the seemingly conflicting results in the existing literature. The ability to reconcile findings from different studies by considering various previously unaccounted for parameters highlights the substantial contribution of our study.

## Supplementary Material

Supplementary Material

## Data Availability

The 1.5T raw data are publicly available athttps://osf.io/94c5t/. The other raw data will be made available by request to A.L.G. (3T) and J.J. (7T). The code used for data analysis is available athttps://github.com/LaSEEB/eeg_fmri_consistency.
