# Supplementary material for: Consistency of resting-state correlations between fMRI networks and EEG band power
Source: Imaging Neurosci (Camb). 2025 Jun 18;3:IMAG.a.37. doi: 10.1162/IMAG.a.37 (PMC12320011; doi:10.1162/IMAG.a.37)
Supplement: Supplementary Material [file imag.a.37_supp.pdf]

Supplementary Materials

Review of EEG-fMRI Correlates

Table S1. Studies that investigate EEG spectral correlates of fMRI signals.

Study	Study Design	EEG Measures	EEG Freq. Bands (Hz)	fMRI Measures	Integration	Results	
						Positive Relationship	Negative Relationship
Goldman et al., 2002	11 subjects<br>Rest, EC<br>3T MR scanner<br>16 EEG channels	Band-specific power TS;<br>HRF conv. (6-s peak);<br>Avg. across T6-O2, O2-P4, T5-O1, O1-P3	α (8-12)	Voxel-wise whole-brain TS	GLM	Insula (LN): α<br>Thalamus: α	Occipital (VN): α<br>ACC (DMN): α<br>Sup. temp. (LangN, AudN): α
Laufs et al., 2003a	15 subjects<br>Rest, EC<br>1.5T MR scanner<br>29 EEG channels	Band-specific power TS;<br>HRF conv. (6-s peak, SPM99);<br>Avg. across O2, O1; O2, O1;<br>C4, C3; F4, F3;	α (8-12)	Voxel-wise whole-brain TS	GLM	N/A	Bilateral inf. parietal (DMN): α<br>Bilateral sup. par./ sup. front. (DAN): α<br>Inf. front. (VAN): α<br>Sup. temp. (LangN, AudN): α
Moosmann et al., 2003	20 subjects<br>Rest, EC<br>1.5T MR scanner<br>29 EEG channels	Band-specific power TS;<br>HRF conv. (6-s peak, SPM99);<br>Avg. across O2, O1	α (8-12)	Voxel-wise whole-brain TS	GLM	N/A	Occipital (VN): α<br>Inf. par. (DMN): α<br>Inf. front. (VAN): α
Laufs et al., 2003b	15 subjects<br>Rest, EC<br>1.5T MR scanner<br>29 EEG channels	Band-specific power;<br>HRF conv. (6-s peak, SPM99);<br>Avg. across O2, O1	θ (4-7)<br>α (8-12)<br>β-1 (13-16)<br>β-2 (17-23)<br>β-3 (24-30)	Voxel-wise whole-brain TS	GLM	PCC, precuneus, mPFC (DMN): β-2; ACC: β-3<br>Temporo-parietal junction (VAN): β-2	PCC (DMN): β-3<br>Bilateral prefrontal and par. (VAN): α Temporo-parietal junction (VAN): β-3
Feige et al., 2005	7 subjects<br>Rest, EC/EO<br>1.5T MR scanner<br>29 EEG channels	Alpha ICs (tICA) power TS;<br>HRF conv. (6-s peak, AFNI);<br>All channels	α (8-12 Hz)	Voxel-wise whole-brain TS	GLM	N/A	EC/EO: Occipital (VN): α (occ.)
Gonçalves et al., 2006	7 subjects<br>Rest, EC<br>1.5T MR scanner<br>19 EEG channels	Band-specific power TS;<br>HRF conv. (6-s peak, AFNI);<br>Avg. across C3–P3, P3–O1, O1–T5, T5–T3, C4–P4, P4–O2, O2–T6, T6–T4	α (8-12)	Voxel-wise whole-brain TS	GLM	Thalamus: α	Precuneus (DMN): α<br>Occipital (VN): α<br>Sup. front. (DAN): α

Laufs et al., 2006	15 subjects<br>Rest, EC<br>1.5T MR scanner<br>29 EEG channels	Band-specific power TS;<br>HRF conv. (6-s peak, SPM99);<br>Avg. across O2, O1	θ (4-7)<br>α (8-12)<br>β (13-30)	Voxel-wise whole-brain TS	GLM	N/A	Bilateral inf. parietal (DMN): α<br>Bilateral sup. parietal/ sup. front. (DAN): α<br>Inf. front. (VAN): α<br>Occipital (VN): α
Mantini et al., 2007	15 subjects<br>Rest, EC<br>1.5T MR scanner<br>32 EEG channels	Band-specific power TS;<br>HRF conv. (6-s peak);<br>Avg. across all channels	δ (1-4)<br>θ (4-8)<br>α (8-13)<br>β (13-30)<br>γ (30-50)	sICA – IC TS (DMN)	Univariate Pearson's correlation	DMN: δ, θ, α, β, γ	VN: δ, θ, α, β, γ<br>SMN: δ, θ, α, β, γ<br>DAN: δ, θ, α, β, γ
DiFrancesco et al., 2008	20 subjects<br>Rest, EC<br>1.5T MR scanner<br>64 EEG channels	Band-specific power TS;<br>HRF conv. (6-s peak, SPM5);<br>Avg. across P3-O1, P4-O2, P7-O1, P8-O2	α (8-13)	Voxel-wise whole-brain TS	GLM	ACC (DMN, Limbic): α<br>Somatomotor (SMN): α<br>Thalamus, cerebellum: α	Occipital (VN): α<br>Dorsal prefrontal (VAN): α
Scheeringa et al., 2008	20 subjects<br>Rest, EO<br>1.5T MR scanner<br>29 EEG channels	Theta frontal midline IC TS;<br>HRF conv. (6-s peak, SPM5);<br>All channels	θ (2-9)	Voxel-wise whole-brain TS	GLM	N/A	mPFC, ACC, precuneus, bilateral inf. parietal (DMN): θ<br>Bilateral inf. front. (VAN): θ
de Munck, 2009	16 subjects<br>Rest, EC<br>1.5T MR scanner<br>64 EEG channels	Band-specific power TS;<br>HRF conv. (data-driven);<br>Avg. across O1, O2, POz, PO3, PO4, PO7, PO8, P5-PO3, P6-PO4	δ (0.1-4)<br>θ (4.5-8)<br>α (8.5-12)<br>β (12.5-36)<br>γ (36.5-100)	Voxel-wise whole-brain TS	GLM	Thalamus: α, β	Occipital (VN): α, β
Britz et al., 2010	9 subjects<br>Rest, EC<br>3T MR scanner<br>64 EEG channels	TS of 4 microstates;<br>HRF conv. (6-s peak, SPM);<br>All channels	N/A	Voxel-wise whole-brain TS	GLM	SN: Microstate 3 (neg. front; pos. back)	AudN: Microstate 1 (neg. front-right; pos. back-left)<br>VN: Microstate 2 (neg. front-left; pos. back-right)<br>DAN: Micsrostate 4 (neg. front. centr., pos. rest)
Jann et al., 2010	14 subjects<br>Rest, EC<br>3T MR scanner<br>92 EEG channels	Band-specific power TS;<br>4-6s delay;<br>All channels	δ (1.0-3.5)<br>θ-1 (3.5-6.25)<br>θ-2 (6.25-8.2)<br>α-1 (8.2-10.5)<br>α-2 (10.5-14.0)<br>β-1(14.0-18.75)<br>β-2 (18.75-21.88)<br>β-3 (21.88-30)	sICA – IC TS (VN, SMN, FPN, DMN)	Univariate covariance	VN: δ, θ (occ.);<br>SMN: δ (cent.)<br>FPN: α-1, α-2, β-1, β-2 (occ.)<br>DMN: α-1 (central); α-2 (par-occ.); β-1 (par.)	VN: α-1, α-2, β-1, β-2 (occ., par.)<br>SMN: : α-1, α-2 (cent-par.), β-1, β-2 (cent-par.)<br>FPN: α-2 (front.), θ (cent-occ.)<br>DMN: δ, θ-1, θ-2 (fronto-cent., par-occ.)
Musso et al., 2010	11 subjects<br>Rest, EC<br>3T MR scanner<br>30 EEG channels	TS of 7 microstates (group);<br>HRF conv. (variable peak);<br>All EEG channels; source-space (sLORETA)	N/A	Voxel-wise whole-brain TS	GLM	VN, DMN: Microstate 4	N/A

Sadaghiani et al., 2010	26 subjects<br>Rest, EC<br>3T MR scanner<br>62 EEG channels	Band-specific power TS;<br>HRF conv. (6-s peak, SPM);<br>All channels	low α (7-10)<br>upper α (9-12)<br>low β (15-18)<br>broad β (17-24)	Seed-based regression RSN TS (TAN, DAN)	Multivariate regression	TAN: upper α, broad β	DAN: low α, low β
Tagliazucchi, 2012	15 subjects<br>Rest, EC<br>3T MR scanner<br>30 EEG channels	Band-specific power TS;<br>All channels	δ (0.4-4)<br>θ (4-8)<br>α (8-12)<br>σ (12-15)<br>β (15-30)<br>γ (30-60)	TS of dFC (AAL, SW Pearson's correlation);<br>TS of graph metrics	Univariate Pearson’s correlation	dFC in front., precuneal, temp. areas: γ<br>dFC avg. path length: α (front., centr.)	dFC in thalamus, association areas. α, β
Yuan et al., 2012	9 subjects<br>Rest, EC<br>3T MR scanner<br>126 EEG channels	TS of 13 ICs (tICA);<br>HRF conv. (canonical);<br>All channels	N/A	sICA – IC TS (VN1, VN2, SMN1, SMN2, AudN, L/R AN, FN)	Univariate Pearson’s correlation	MS1: DMN, FN<br>MS2: SMN, AudN<br>MS3: SMN<br>MS4, MS5: R, L AN<br>MS7–12: SMN, VN, AudN<br>MS13: DMN, FN, AN, VN	N/A
Bridwell et al., 2013	25 subjects<br>Rest, EC, EO<br>1.5T MR scanner<br>30 EEG channels	TS of 10 spectral ICs;<br>All channels	δ (1-1.5)<br>θ (2.5-5)<br>α (6.5-13)<br>β (13-29)<br>γ (31.5-35)	sICA - IC TS (56)	BOLD sICs deconvolved with EEG spectral IC TS	Frontal, parietal, temporal, limbic, occipital: δ<br>Frontal, parietal, occipital: γ	Subcortical: δ<br>Frontal, parietal, temporal, limbic, occipital: α<br>Temporal, subcortical, cerebellum: γ
Chang et al., 2013	10 subjects<br>Rest, EC<br>3T MR scanner<br>256 EEG channels	Band-specific power TS;<br>Median across O1, O2, Oz, P3, P4, Pz, C3, C4, F3, F4, Fz	θ (4-7)<br>α (7-13)	TS of pairwise dFC between RSNs (DMN, DAN, SN)	SW univariate Pearson’s correlation	Within DMN connectivity: θ	DMN-DAN connectivity: α
Mayhew et al., 2013	14 subjects<br>Rest, EC<br>3T MR scanner<br>64 EEG channels	Band-specific power TS;<br>HRF conv. (6-s peak, FSL);<br>PO3/PO4, POz, O1/O2, Oz	α (7-13)	Positive/negative BOLD response to a visual stimulus within RSNs	GLM	VN: α	DMN: α
Meyer et al., 2013	12 subjects<br>Rest, EO<br>3T MR scanner<br>30 EEG channels	Band-specific power TS;<br>HRF conv. (6-s peak, SPM5);<br>Avg. across all channels	δ (2-4)<br>θ (4-7)<br>α (8-12)<br>β (12-30)	sICA – IC TS (VN, SMN, FPN, DMN)	Univariate Pearson’s correlation	VN: δ, θ, β<br>SMN: δ, θ<br>FPN: δ<br>DMN: δ, θ	VN: α<br>SMN: α, β<br>FPN: α, β, θ<br>DMN: α, β
Mo et al., 2013	14 subjects<br>Rest, EC, EO<br>3T MR scanner<br>31 EEG channels	Band-specific power TS;<br>HRF conv. (canonical);<br>Avg. across O1, O2, Oz	α (8-12)	Voxel-wise whole-brain TS	GLM	PCC, mPFC, bilateral inferior parietal cortex (DMN): α (EO)	(FPN): α (EO, EC)

Hiltunen et al., 2014	21 subjects<br>Rest, EC<br>1.5T MR scanner<br>32 EEG channels	TS of ISF ICs (tICA);<br>HRF conv. (6-s peak, FSL);<br>All channels	ISF (0.01-0.1)	Voxel-wise whole-brain TS; sICA – IC TS (VN, DMNpcc, DMNvmpf, DAN, SN, S2, M1, ECN)	GLM	VN, DMNpcc, DMNvmpf, ECN	DAN, SN
Neuner et al., 2014	15 subjects<br>Rest, EC<br>3T MR scanner<br>63 EEG channels	Band-specific power TS;<br>TS of ROIs within DMN (source-space, LORETA)	δ (0.5-3.5)<br>θ (4-7)<br>α-1 (7.5-9.5)<br>α-2 (10-12)<br>β-1 (13-23)<br>β-2 (24-34)	sICA – IC TS (DMN)	Multivariate regression	Parahippocampal gyrus (DMN): δ (ACC);<br>SMA (SMN): β-1 (PCC)	N/A
Schwab et al. 2015	14 subjects<br>Rest, EC<br>3T MR scanner<br>92 EEG channels	TS of 6 microstates;<br>HRF conv. (canonical);<br>All channels	δ (1-3.5),<br>θ1 (3.5-6.25)<br>θ2 (6.25-8.2)<br>α1 (8.2-10.5)<br>α2 (10.5-14)<br>β1 (14-18.75)<br>β2 (18.75-21.88)<br>β3 (21.88-30)	Voxel-wise TS in RSNs (DMN, SMN, DAN)	GLM	DMN: MS A, MS D<br>SMN: MS B<br>DAN: MS F	N/A
Yin et al., 2016	36 subjects<br>Rest, EC<br>3T MR scanner<br>31 EEG channels	TS of Mu IC (tICA);<br>HRF conv. (6-s peak, SPM5)<br>All channels	μ (8-12)	Voxel-wise whole-brain TS	GLM	ACC, anterior insula (SN, DMN, LN): μ	Areas within SMN, DAN: μ
Bowman et al., 2017	20 subjects<br>Rest, EC<br>3T MR scanner<br>62 EEG channels	Band-specific power TS;<br>HRF conv. (6-s peak, SPM);<br>Avg. across P3-O1, P4-O2, P7-O1, P8-O2	α (8-13)	sICA – IC TS (subnetworks of DMN)	Univariate Pearson's correlation	DMN: α (PCC + mPFC + precuneus)	DMN: α (PCC + parietal; sup. front. + ACC; medial frontal + bilateral temporal)
Marawar et al., 2017	14 subjects<br>Rest, EC<br>3T MR scanner<br>32 EEG channels	Band-specific power TS<br>HRF conv. (6-s peak, FSL);<br>F7, T7, TP9, F8, T8, TP10 (avg. at each channel trio (left/right))	δ (1-4)<br>θ (4.1-8)	Voxel-wise whole-brain TS	GLM	Precuneus (DMN): δ (left, right); dorsal mPFC (DMN) (left): θ	Precuneus (DMN): θ (left, right); dorsal mPFC (left): δ
Mayhew and Bagshaw, 2017	32 subjects<br>Rest, EO<br>3T MR scanner<br>62 EEG channels	Band-specific power TS<br>HRF convolution (6-s peak, FSL);<br>Avg. across PO3, PO4, POz, O1, O2, Oz	α (8-13)	Voxel-wise whole-brain TS	SL univariate PC (dynamic);<br>GLM (static)	PCC, mPFC (DMN): α (static) (~50% epochs, dynamic)	
Tsuchimoto et al., 2017	19 subjects<br>Rest, EO<br>1.5T MR scanner<br>63 EEG channels	SMR TS (power of C3 in α and β);<br>HRF conv. (canonical)<br>C3	α (7-11)<br>β (12-30)	Voxel-wise whole-brain TS	Univariate Pearson's correlation	N/A	Pericentral area (SMN): α, β

Allen et al., 2018	23 subjects<br>Rest, EO, EC<br>1.5T MR scanner<br>32 EEG channels	Time-frequency spectrum;<br>Cz, O1, O2	δ (1-4)<br>θ (4-8)<br>α (8-12)	dFNC states (dynamic pairwise PC between ICN TS + k-means) (DMN, VN, CCN, SMN, AudN, SCN, CBN)	EEG power spectra from each TR segregated into groups based on concurrent FNC state vectors	S1: Weak α, minimal δ/θ<br>S2: Central α peak<br>S3: Strong occipital α<br>S4: Broadened α, slow oscillations (< 7 Hz)<br>S5: Dominant δ/θ, occipital α desynchronization	
Lamos et al., 2018	50 subjects<br>Rest, EC<br>1.5T MR scanner<br>30 EEG channels	Spatial-temporal-spectral patterns from PARAFAC (PRFC2, PRFC7, PRFC10);<br>6-s delay;<br>All channels	θ (~4)<br>α (8-10)<br>β (13-30)<br>γ (>30)	TS of dFC between LSBNs (VN, SMN, SAN, FPN, DMN, AudN); BNCS states (PS, NS, D)	mANOVA: PARAFAC patterns (PRFC2, PRFC7, PRFC10), BNCS states (PS, NS, D) → Band-specific power, dFC states	PRFC2: α, β → D in AudN, DAN, SMN; γ → D in AudN, DAN, SMN, FPN;<br>PRFC7: θ, α → PS between DMN and VN;<br>PRFC10: θ → PS ↔ NS transitions in AudN, FPN, DAN; α → PS ↔ NS transitions in AudN, FPN, DAN	
Portnova et al., 2018	25 subjects<br>Rest, EC<br>3T MR scanner<br>32 EEG channels	TS of linear (PSD, mALP, fALP) and non-linear features (EMF, RAT, HFD);<br>HRF conv. (6-s peak, SPM8);<br>All channels	PSD: δ (2-4), α (8-12), β (16-20);<br>HFD: 2-20, α (10-12)	Voxel-wise whole-brain TS	GLM	DMN: δ PSD, fALP<br>SMN, VN: HFD	N/A
Prestel, 2018	30 subjects<br>Rest, EC<br>1.5T MR scanner<br>30 EEG channels	TS of ICs (AMICA);<br>HRF conv. (6-s peak, FSL);<br>All channels	δ (1-4)<br>θ (4-8)<br>α1 (8-10)<br>α2 (10-12)<br>β1 (12-20)<br>β2 (20-30)<br>γ (35-45)	Voxel-wise whole-brain TS; sICA – IC TS (DMN)	GLM	DMN: β1	DMN: δ, θ, γ
Labounek et al., 2019	50 subjects<br>Rest, EC<br>1.5 MR scanner<br>30 EEG channels	Spatial-spectral patterns from group ICA;<br>HRF conv. (data-driven);<br>All channels	δ1, δ2, δ3, δ4 (0-4)<br>θ1, θ2, θ3, θ4, θ5 (4-8)<br>α1, α2, α3 (8-12)<br>β1, β2 (12-30)	Voxel-wise whole-brain TS	GLM<br>Coefs. of HRF (+dHRF) convolved regressors used to estimate HRF peak	DMN: α3;<br>Basal Ganglia Net.: δ	
Rajkumar et al., 2021	10 subjects<br>Rest, EC<br>3T MR scanner<br>31 EEG channels	GFP metrics of 4 microstates;<br>All channels	N/A	ReHo, DC and fALFs within DMN voxels	Spearman correlation GFP of MS maps fMRI metrics	DC: Microstate C	N/A
Phadikar et al., 2025	90 subjects<br>Rest, EO<br>3T MR scanner<br>64 EEG channels	Band-specific power TS;<br>HRF conv. (5-s peak);<br>Fz, Cz, Pz	δ (0.5-4)<br>θ (4-8)<br>α (8-13)<br>β (13–30)	scICA - IC TS (14 RSNs)	SW univariate Pearson's correlation	VIS-P: α (Cz, Pz)<br>MTR-P: δ (Cz, Fz), θ (Cz, Pz), β (Cz, Fz, Pz)<br>CER: δ (Pz), θ (Cz, Fz, Pz), α (Cz, Fz), β (Cz, Fz)<br>DMN-A: δ (Fz, Pz)	N/A

Acronym list: ACC (Anterior Cingulate Cortex), AMICA (Adaptive Mixture ICA), ATN (Dorsal Attention Network), BNCS (Between-Network Connectivity States), CER (Cerebellar Network), DC (Degree Centrality), DMN (Default Mode Network), DMN-A/P (Anterior/Posterior Default Mode Network), dFC (Dynamic Functional Connectivity), dHRF (temporal derivative of HRF), EC (Eyes Closed), EEG (Electroencephalography), EMF (signal Envelope Mean Frequency), EO (Eyes Open), FC (Functional Connectivity), fALFF (fractional Amplitude of Low-Frequency Fluctuations), fALP (frequency of Alpha Peak), fMRI (functional

Magnetic Resonance Imaging), FPN (Frontoparietal Network), FPN-L/R (Left/Right Frontoparietal Network), FRNT (Frontal Network), GFP (Global Field Power), GLM (General Linear Model), HFD (Higuchi's Fractal Dimension), HRF (Hemodynamic Response Function), IC (Independent Component), ICA (Independent Component Analysis), LangN (Language Network), LORETA (Low-Resolution Electromagnetic Tomography), LSBNs (Large-Scale Brain Networks), mALP (magnitude of Alpha Peak), mANOVA (multivariate Analysis of Variance), MS (Microstate), MTR-P/S (Primary/Secondary Somatomotor Network), PARAFAC (Parallel Factor Analysis), PCC (Posterior Cingulate Cortex), PCu (Precuneus), PRFC (PARAFAC pattern), PSD (Power Spectral Density), ReHO (Regional Homogeneity), RSN (Resting-State Network), scICA (Spatially Constrained ICA), sICA (Spatial ICA), SMN (Somatomotor Network), SMR (Sensorimotor Rhythm), SN (Salience Network), STG (Superior Temporal Gyrus), TAN (Tonic Alertness Network), TEMP (Temporal Network), tICA (Temporal ICA), VAN (Ventral Attention Network), VIS-P/S (Primary/Secondary Visual Network), VN (Visual Network).

fMRI Resting State Network Maps

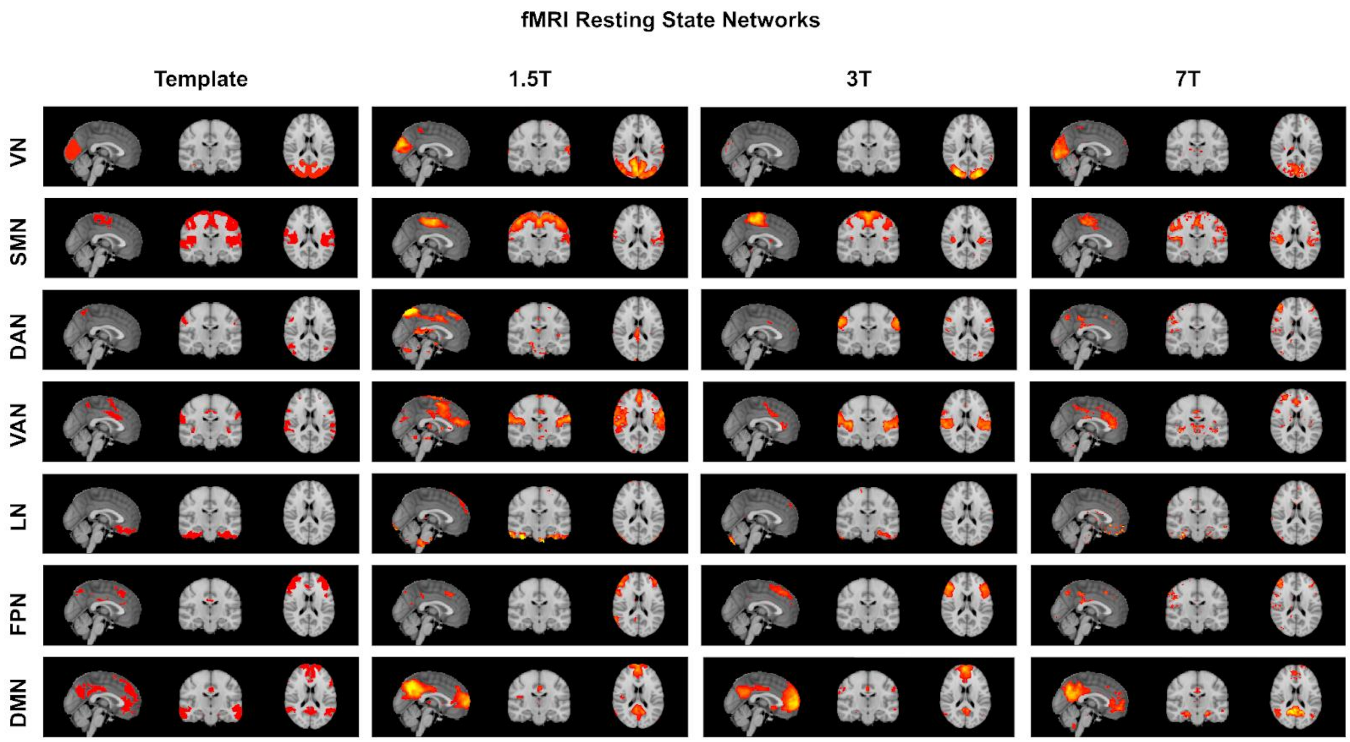

Fig. S1. fMRI Resting State Network Maps. Spatial maps of each of the 7 canonical Resting State Networks (RSNs). From left to right: template maps (Yeo et al., 2011), group Independent Component (IC) maps obtained through group Independent Component Analysis (ICA) for three independent datasets, obtained at 1.5T, 3T and 7T, respectively. Statistical maps obtained in FSL's FSLeyes. Sagittal, coronal and transverse views, thresholded at Z=3.

EEG Band-specific Power Spatial Maps

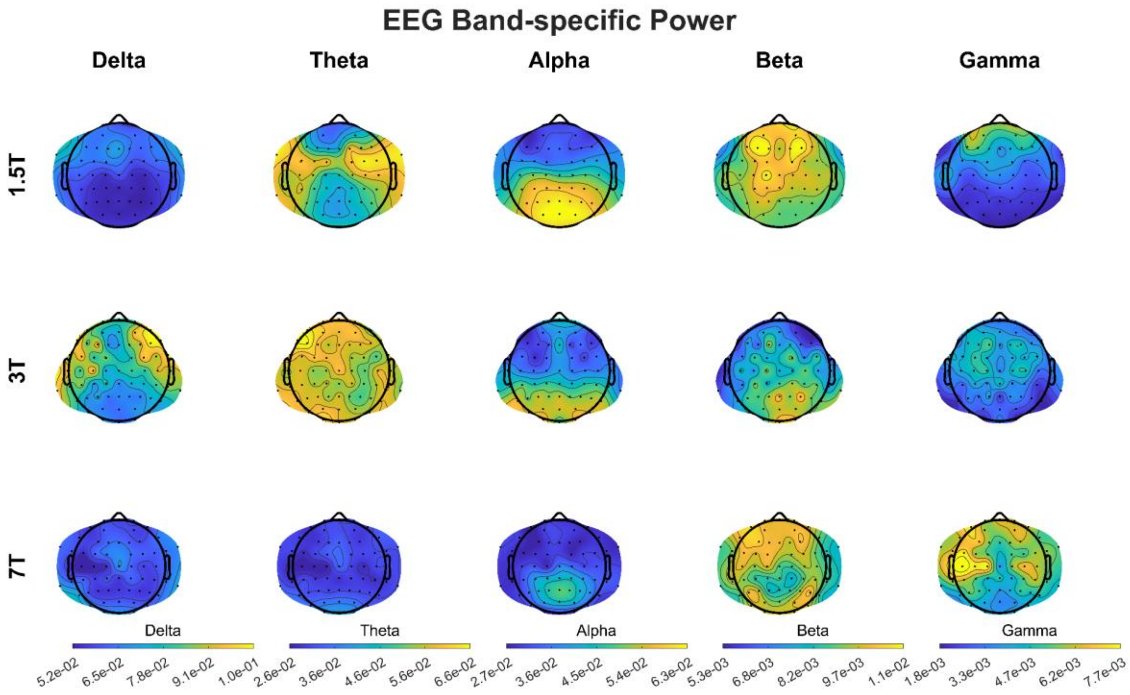

Fig. S2. EEG Band-specific Power Spatial Maps. Spatial maps of the average, across time and subjects, relative power at each frequency band: delta (2-4Hz), theta (5-7Hz), alpha (8-12Hz), beta (15-29Hz), gamma (30-60Hz). Topographies corresponding to three independent datasets, obtained at 1.5T, 3T and 7T, respectively.

Hemodynamic Response Functions

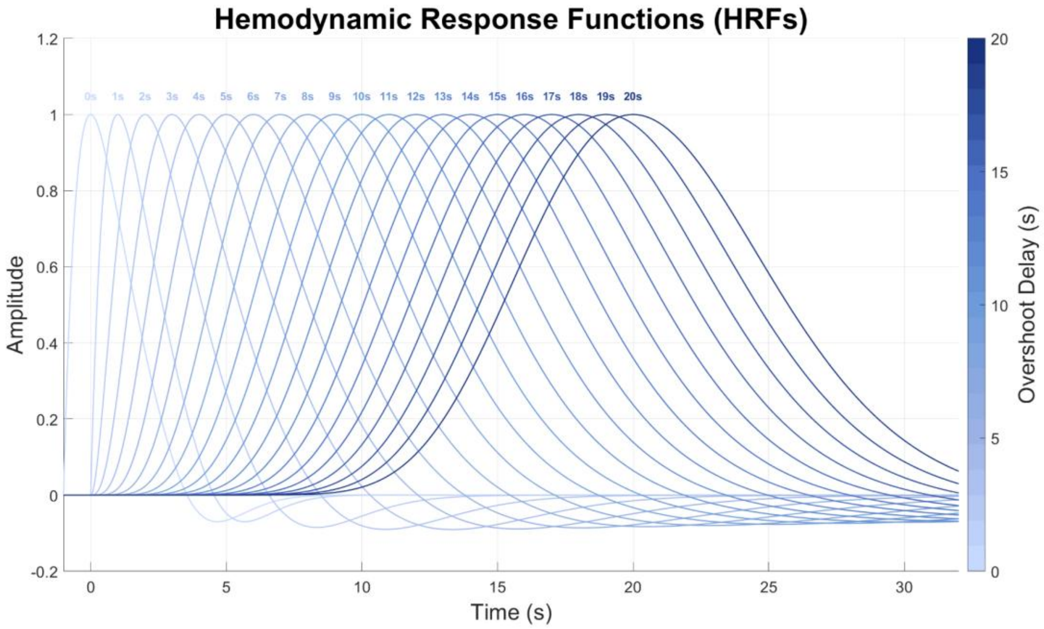

Fig. S3 Time-series of Hemodynamic Response Functions (HRFs). Time-series of 32-s HRFs, computed for overshoot delays (relative to onset) from 0 to 20 s in steps of 1 s. The HRFs are modelled by the linear combination of two gamma functions, one modelling the positive response peak and the other the negative post-stimulus undershoot. The gamma function parameters are adjusted to produce the desired overshoot delays, and the undershoot delay and dispersion of both overshoot and undershoot are linearly scaled in relation to the overshoot delay to preserve the HRF shape.

EEG-fMRI Correlation Spatial Maps

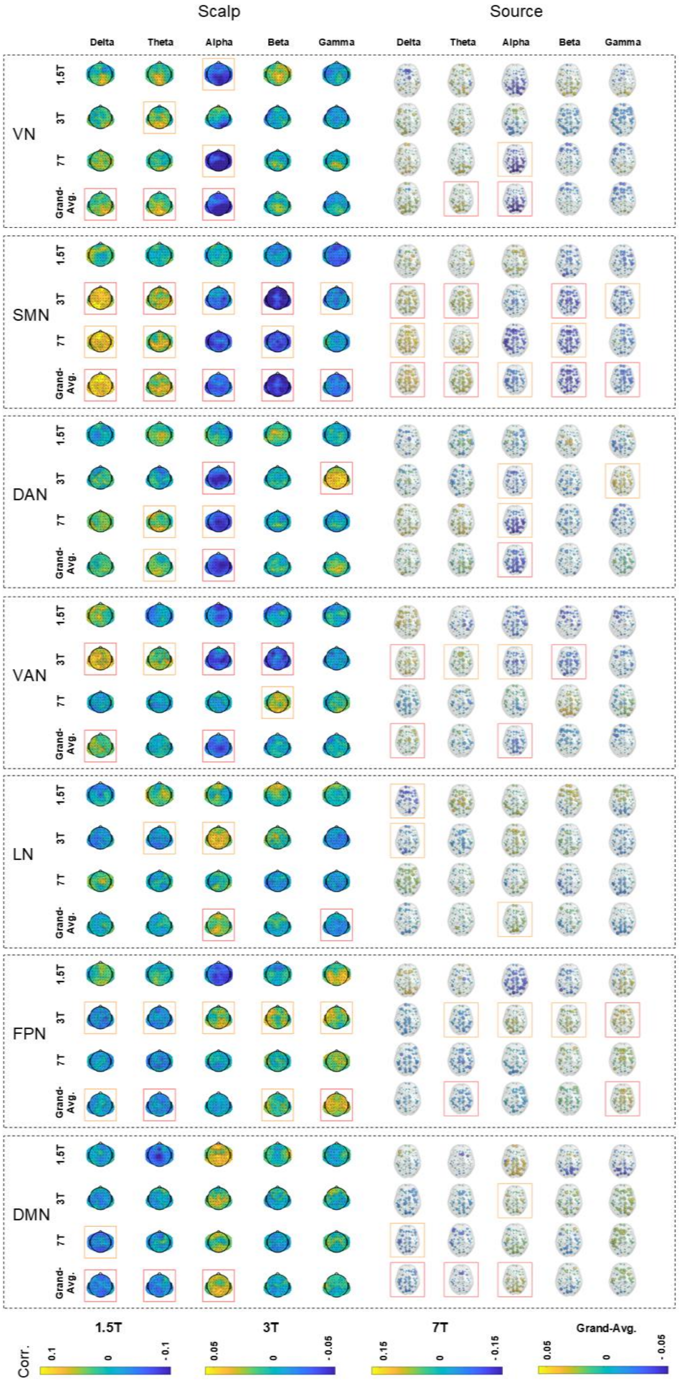

Fig. S4. Spatial maps of EEG-fMRI temporal correlations with a 2s HRF delay. The subject-averaged spatial maps of EEG-fMRI temporal correlations obtained using the canonical HRF with a 2-s overshoot delay are shown for each fMRI RSN (rows) and EEG frequency-band (columns) in both scalp (channels) and source (regions of the Desikan atlas) spaces (left-right). Results are presented for each of the individual datasets (1.5T, 3T, and 7T) and for the grand-average across datasets. Boxes highlight where the correlations are significantly different from zero ($p < 0.05$) based on one-sample t-tests across subjects: orange boxes indicate uncorrected results, and red boxes indicate that remain significant False Discovery Rate correction.

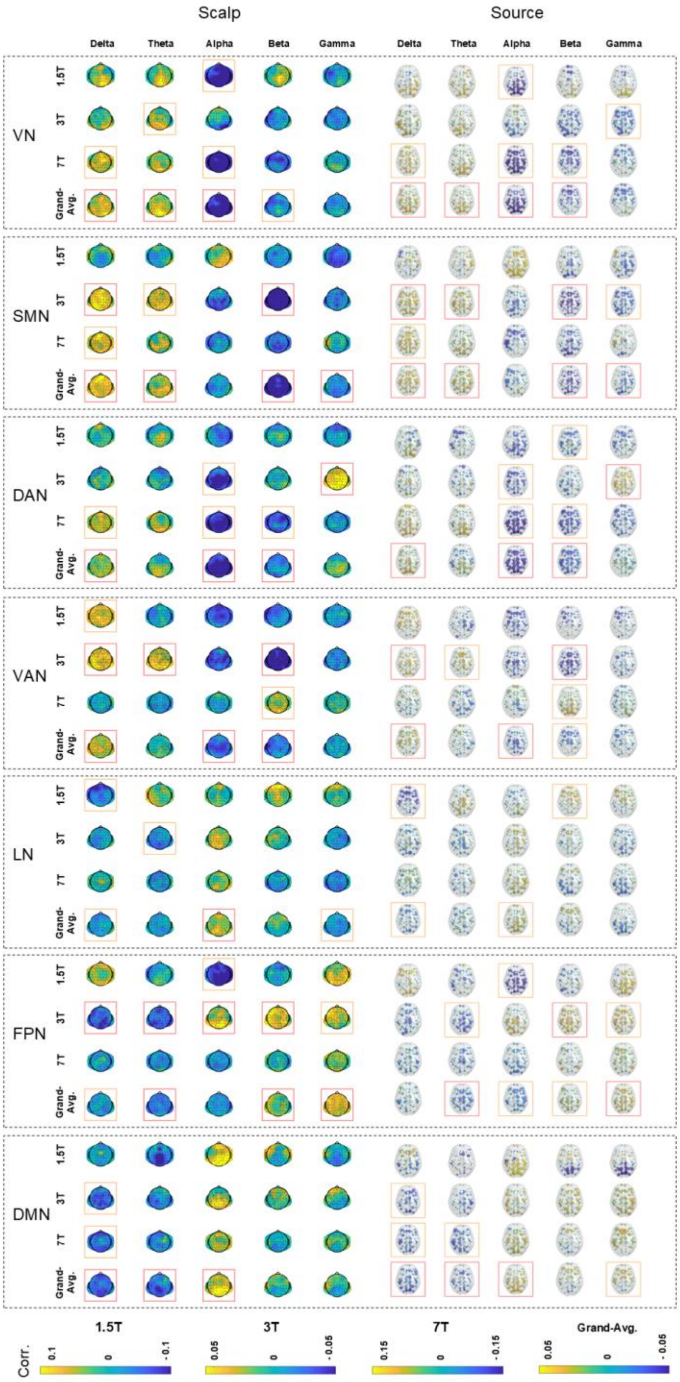

Fig. S5. Spatial maps of EEG-fMRI temporal correlations with a 4s HRF delay. The subject-averaged spatial maps of EEG-fMRI temporal correlations obtained using the canonical HRF with a 4-s overshoot delay are shown for each fMRI RSN (rows) and EEG frequency-band (columns) in both scalp (channels) and source (regions of the Desikan atlas) spaces (left-right). Results are presented for each of the individual datasets (1.5T, 3T, and 7T) and for the grand-average across datasets. Boxes highlight where the correlations are significantly different from zero ($p < 0.05$) based on one-sample t-tests across subjects: orange boxes indicate uncorrected results, and red boxes indicate that remain significant False Discovery Rate correction.

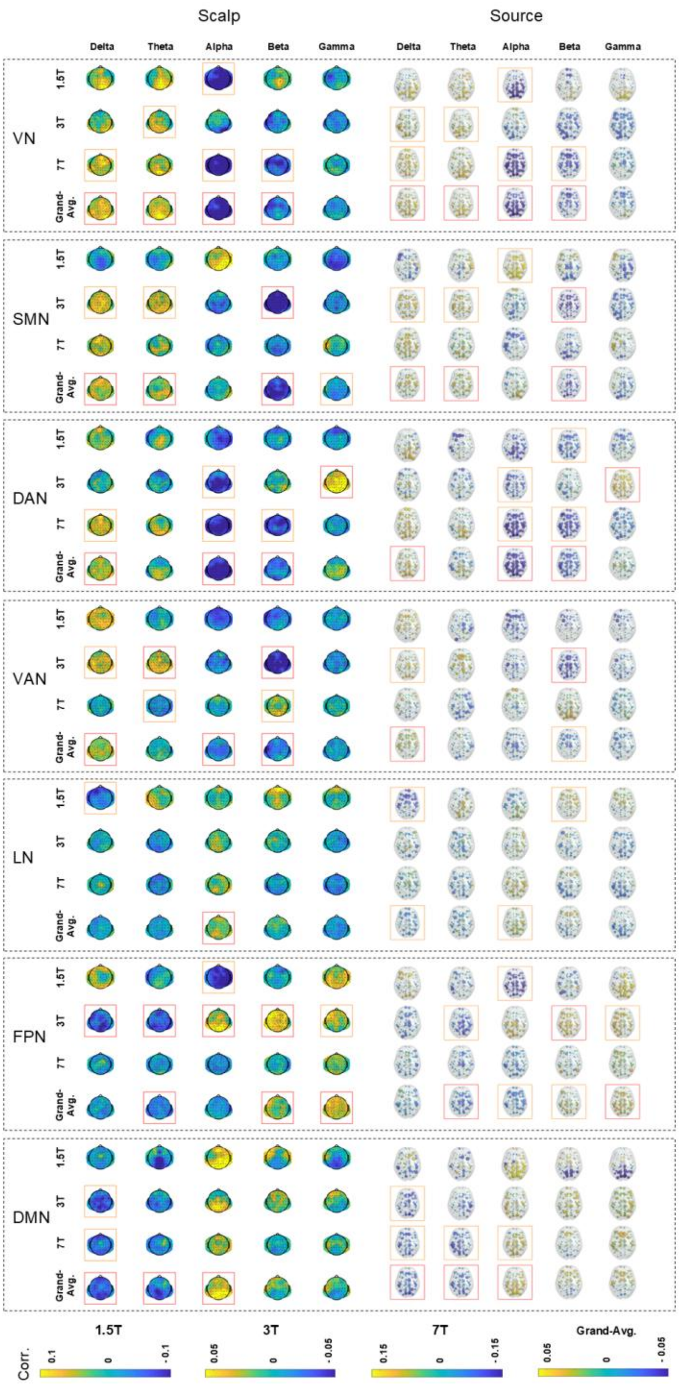

Fig. S6. Spatial maps of EEG-fMRI temporal correlations with a 5s HRF delay. The subject-averaged spatial maps of EEG-fMRI temporal correlations obtained using the canonical HRF with a 5-s overshoot delay are shown for each fMRI RSN (rows) and EEG frequency-band (columns) in both scalp (channels) and source (regions of the Desikan atlas) spaces (left-right). Results are presented for each of the individual datasets (1.5T, 3T, and 7T) and for the grand-average across datasets. Boxes highlight where the correlations are significantly different from zero ($p < 0.05$) based on one-sample t-tests across subjects: orange boxes indicate uncorrected results, and red boxes indicate that remain significant False Discovery Rate correction.

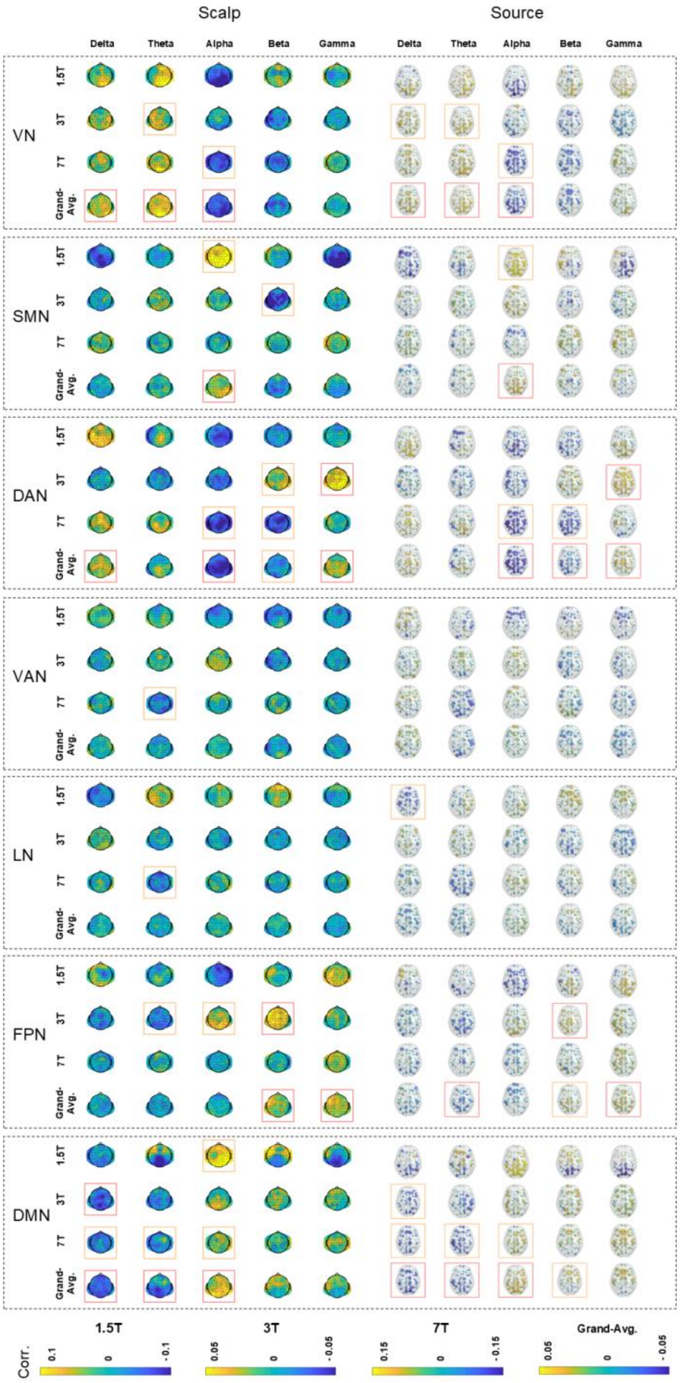

Fig. S7. Spatial maps of EEG-fMRI temporal correlations with a 8s HRF delay. The subject-averaged spatial maps of EEG-fMRI temporal correlations obtained using the canonical HRF with a 8-s overshoot delay are shown for each fMRI RSN (rows) and EEG frequency-band (columns) in both scalp (channels) and source (regions of the Desikan atlas) spaces (left-right). Results are presented for each of the individual datasets (1.5T, 3T, and 7T) and for the grand-average across datasets. Boxes highlight where the correlations are significantly different from zero ($p < 0.05$) based on one-sample t-tests across subjects: orange boxes indicate uncorrected results, and red boxes indicate that remain significant False Discovery Rate correction.

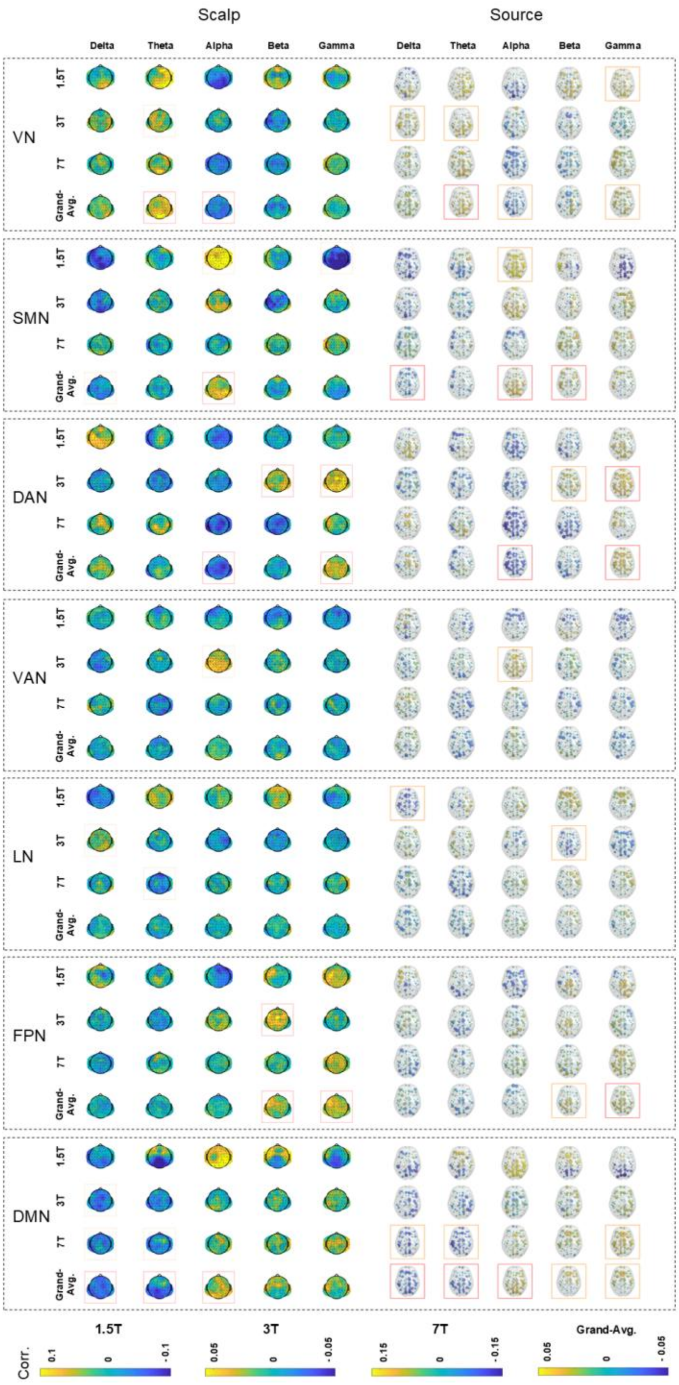

Fig. S8. Spatial maps of EEG-fMRI temporal correlations with a 10s HRF delay. The subject-averaged spatial maps of EEG-fMRI temporal correlations obtained using the canonical HRF with a 10-s overshoot delay are shown for each fMRI RSN (rows) and EEG frequency-band (columns) in both scalp (channels) and source (regions of the Desikan atlas) spaces (left-right). Results are presented for each of the individual datasets (1.5T, 3T, and 7T) and for the grand-average across datasets. Boxes highlight where the correlations are significantly different from zero ($p < 0.05$) based on one-sample t-tests across subjects: orange boxes indicate uncorrected results, and red boxes indicate that remain significant False Discovery Rate correction.

Spatially Averaged EEG-fMRI Correlations

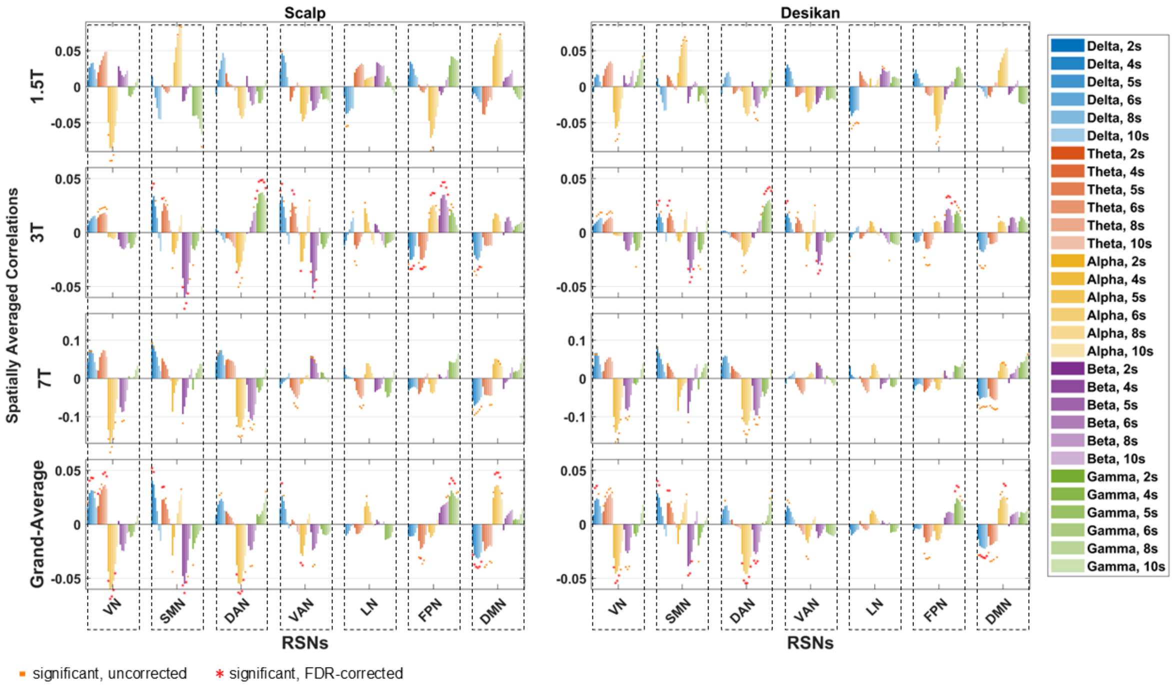

Fig. S9. EEG-fMRI spatially averaged temporal correlations. The bar plots show spatially averaged EEG-fMRI correlations, averaged across subjects, for each dataset (1.5T, 3T, and 7T), and for the grand-average across all datasets, separately for scalp and source EEG spaces. Results for the seven RSNs are displayed from left to right, for each EEG frequency-band (δ, θ, α, β, γ; colors) and HRF delay (2, 4, 5, 6, 8, and 10 seconds; hues). Significance was determined using one-sample t-tests against zero ($p < 0.05$): orange dots mark uncorrected results, and red asterisks mark results that remain significant after False Discovery Rate correction.

Effect of the Number of Subjects on EEG-fMRI Correlations

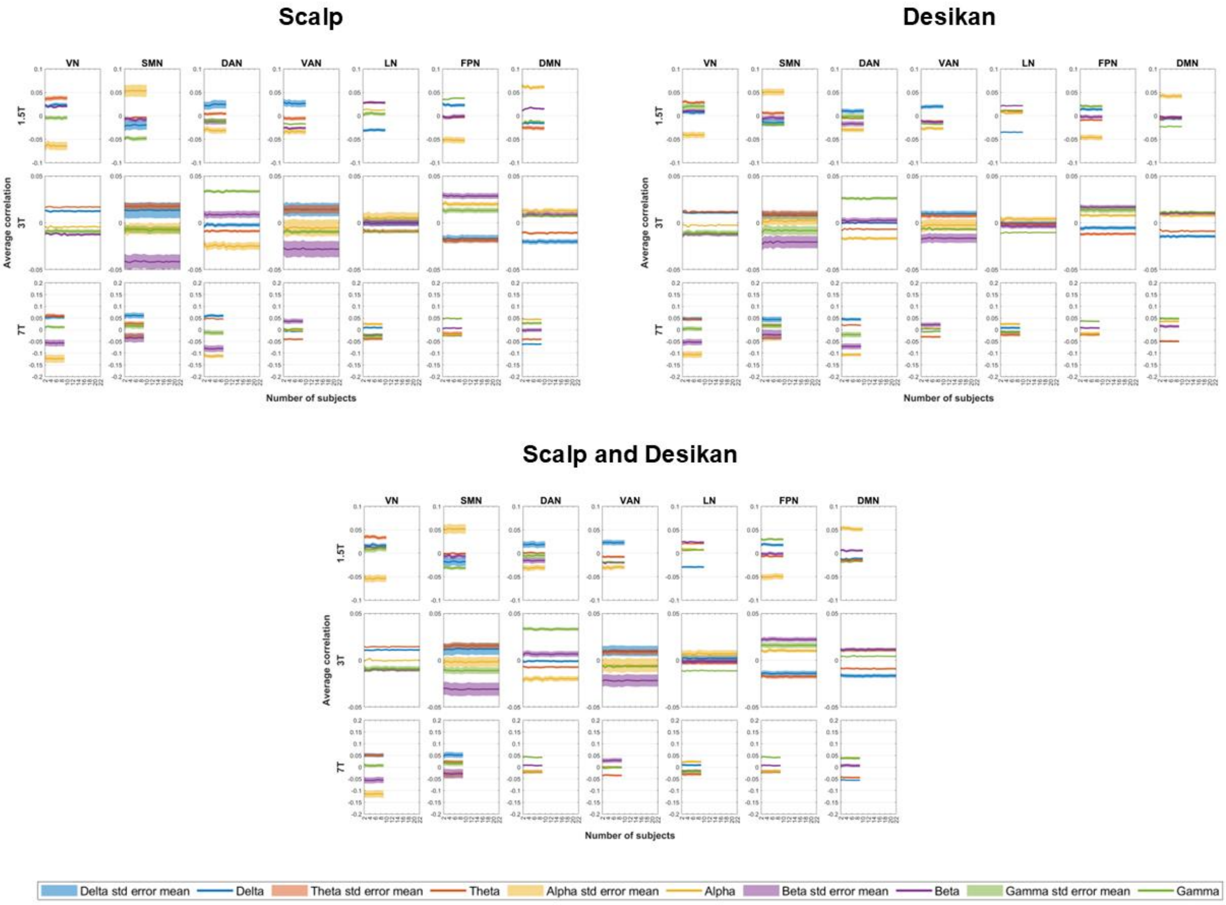

Fig. S10. Effect of the number of subjects on EEG-fMRI correlations. Impact of increasing number of subjects on the EEG-fMRI spatially averaged temporal correlations (averaged across subjects). Top left panel: EEG scalp data; Top right panel: EEG source-space data; Bottom panel: data pooled across both scalp and source EEG spaces to derive average correlation values. Distinct colors denote EEG band-power across across δ, θ, α, β, and γ frequency-bands, with shaded areas indicating the standard mean error across a set of HRF delays (2, 4, 5, 6, 8, and 10s). Rows correspond to each EEG-fMRI dataset (1.5T, 3T, and 7T), while columns correspond to the seven canonical fMRI RSNs. For each dataset, subjects were randomly sampled (ranging from 1 to n subjects, over 5000 iterations) prior to computing the average temporal correlations values.

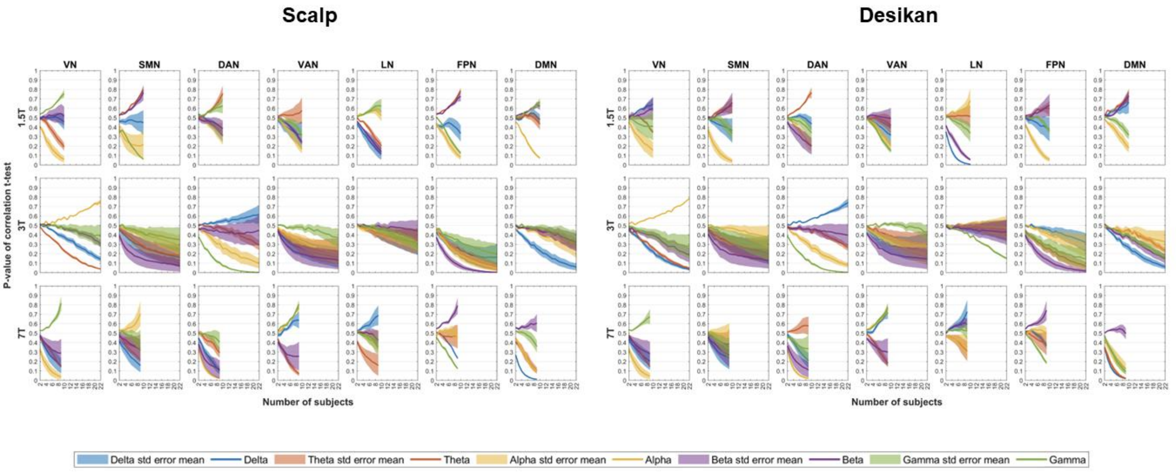

Fig. S11. Effect of the number of subjects on the significance of EEG-fMRI correlations. Impact of increasing number of subjects on the p-value of the t-statistics derived from EEG-fMRI spatially averaged temporal correlations. On the left: EEG scalp data; on the right: EEG source-space data. Distinct colors denote EEG band-power across across δ, θ, α, β, and γ frequency-bands, with shaded areas indicating the standard mean error across a set of HRF delays (2, 4, 5, 6, 8, and 10s). Rows correspond to each EEG-fMRI dataset (1.5T, 3T, and 7T), while columns correspond to the seven canonical fMRI RSNs. For each dataset, subjects were randomly sampled (ranging from 1 to n subjects, over 5000 iterations) prior to computing the t-stat values.

Effect of the Scan Duration on EEG-fMRI Correlations

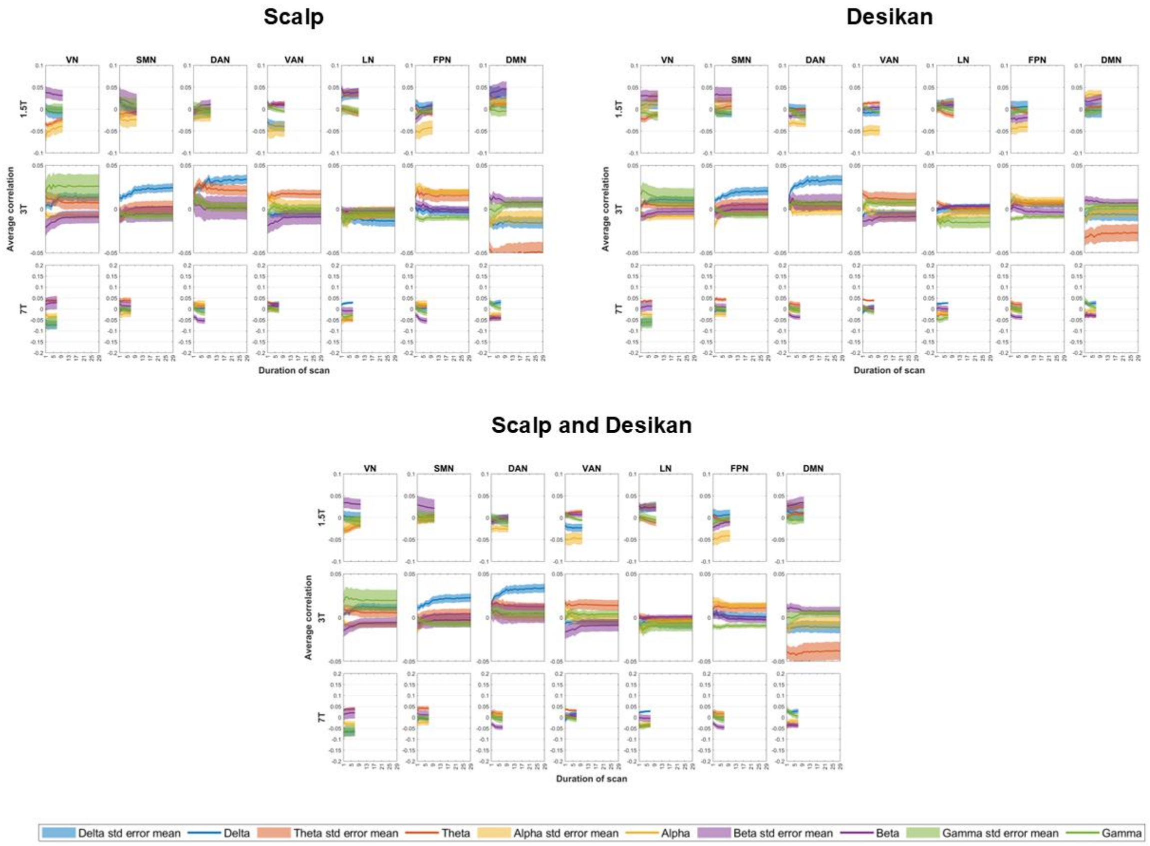

Fig. S12. Effect of the scan duration on EEG-fMRI correlations. Impact of increasing scan duration on the EEG-fMRI spatially averaged temporal correlations (averaged across subjects). Top left panel: EEG scalp data; Top right panel: EEG source-space data; Bottom panel: data pooled across both scalp and source EEG spaces to derive average correlation values. Distinct colors denote EEG band-power across across δ, θ, α, β, and γ frequency-bands, with shaded areas indicating the standard mean error across a set of HRF delays (2, 4, 5, 6, 8, and 10s). Rows correspond to each EEG-fMRI dataset (1.5T, 3T, and 7T), while columns correspond to the seven canonical fMRI RSNs. For each dataset, segments of data were randomly selected (ranging from 1 to n consecutive minutes, over 5000 iterations) prior to computing the average temporal correlations values.

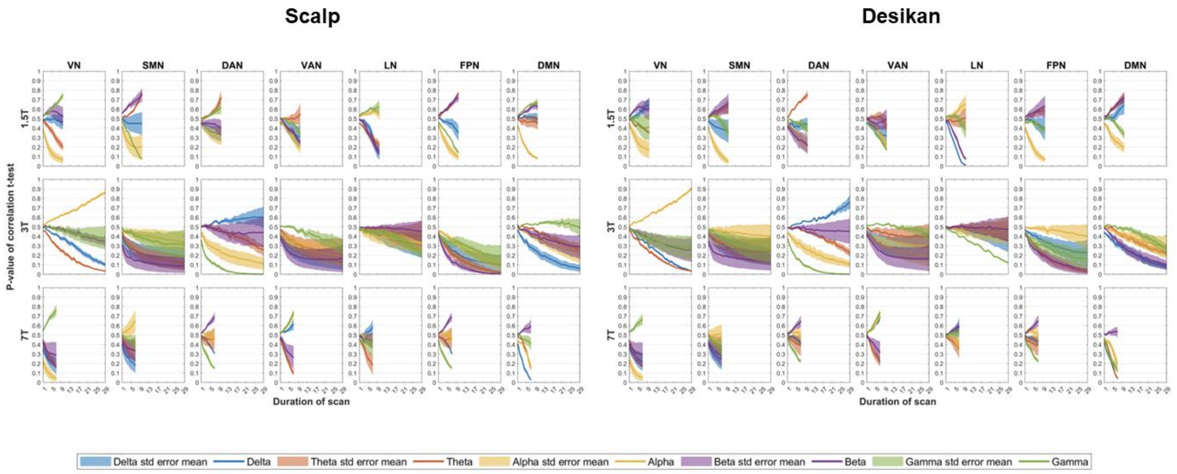

Fig. S13. Effect of the scan duration on the significance of EEG-fMRI correlations. Impact of increasing scan duration on the p-value of the t-statistics derived from EEG-fMRI spatially averaged temporal correlations. On the left: EEG scalp data; on the right: EEG source-space data. Distinct colors denote EEG band-power across across δ, θ, α, β, and γ frequency-bands, with shaded areas indicating the standard mean error across a set of HRF delays (2, 4, 5, 6, 8, and 10s). Rows correspond to each EEG-fMRI dataset (1.5T, 3T, and 7T), while columns correspond to the seven canonical fMRI RSNs. For each dataset, segments of data were randomly selected (ranging from 1 to n consecutive minutes, over 5000 iterations) prior to computing the temporal correlations and t-stat values.

EEG-fMRI Correlations Across Extended HRF delays

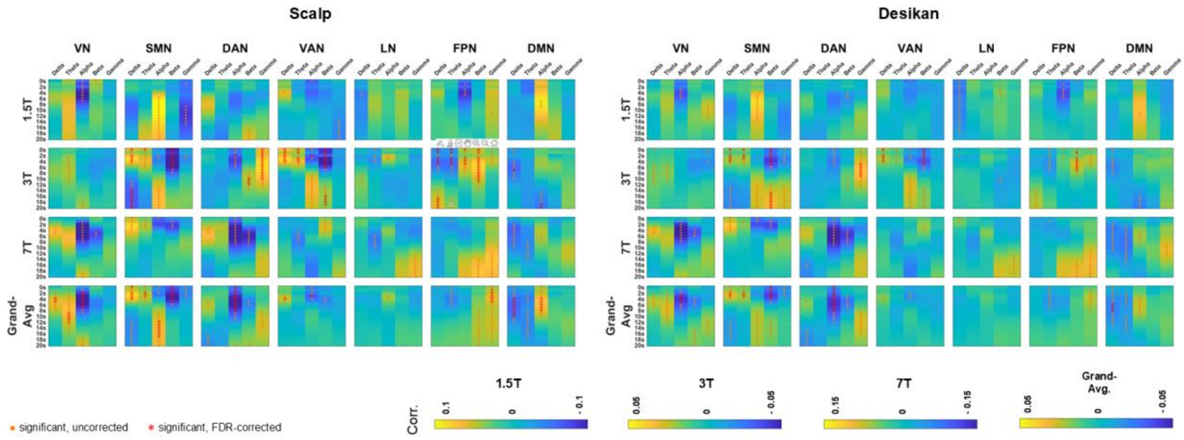

Fig. S14. EEG-fMRI spatially averaged temporal correlations across an extended range of HRF delays. The heatmaps show spatially averaged EEG-fMRI correlations, averaged across subjects, for each dataset (1.5T, 3T, and 7T), and for the grand-average across all datasets, separately for scalp and source spaces. Each subplot corresponds to a particular dataset or the grand-average and to one of the seven RSNs. Within each subplot, rows represent different HRF delays (ranging from 0 to 20 seconds), and columns represent different EEG frequency bands (δ, θ, α, β, γ). Significant correlations, determined by one-sample t-tests against zero ($p < 0.05$), are indicated by orange dots for uncorrected results and red asterisks for False Discovery Rate-corrected results.
